# Possible clinical effects of molecular hydrogen (H_2_) delivery during hemodialysis in chronic dialysis patients: Interim analysis in a 12 month observation

**DOI:** 10.1371/journal.pone.0184535

**Published:** 2017-09-13

**Authors:** Masaaki Nakayama, Noritomo Itami, Hodaka Suzuki, Hiromi Hamada, Naoyuki Osaka, Ryo Yamamoto, Kazumasa Tsunoda, Hirofumi Nakano, Kimio Watanabe, Wan-Jun Zhu, Yukio Maruyama, Hiroyuki Terawaki, Shigeru Kabayama, Ryoichi Nakazawa, Mariko Miyazaki, Sadayoshi Ito

**Affiliations:** 1 United Centers for Advanced Research and Translational Medicine, Center for Advanced and Integrated Renal Science, Tohoku University, Sendai, Japan; 2 Research Division of Chronic Kidney Disease and Dialysis Treatment, Tohoku University Hospital, Sendai, Japan; 3 Department of Nephrology and Hypertension, Fukushima Medical University, Fukushima, Japan; 4 Kidney Center, Nikko-Memorial Hospital and Higashi Muroran Satellite Clinic, Muroran, Japan; 5 Horai-Higashi Clinic, Fukushima, Japan; 6 Gumyoji-Jin Clinic, Yokohama, Japan; 7 Tateishi-Jin Clinic, Tokyo, Japan; 8 Noboribetsu-memorial Hospital, Noboribetsu, Japan; 9 Dialysis center, Kashima Hospital, Iwaki, Japan; 10 Trim Medical Institute Co., Ltd., Osaka, Japan; 11 Department of Nephrology and Hypertension, The Tokyo Jikei University School of Medicine, Tokyo, Japan; 12 Tokatsu Clinic Mirai, Matsudo, Japan; 13 Division of Blood purification, Tohoku University Hospital, Sendai, Japan; Universidade Estadual Paulista Julio de Mesquita Filho, BRAZIL

## Abstract

**Background and aim:**

It is supposed that enhanced oxidative stress and inflammation are involved with the poor clinical outcomes in patients on chronic dialysis treatment. Recent studies have shown that molecular hydrogen (H_2_) is biologically active as an anti-inflammatory agent. Thus, we developed a novel hemodialysis (E-HD) system which delivers H_2_ (30 to 80 ppb)-enriched dialysis solution, to conduct a prospective observational study (UMIN000004857) in order to compare the long-term outcomes between E-HD and conventional-HD (C-HD) in Japan. The present interim analysis aimed to look at potential clinical effects of E-HD during the first 12 months observation.

**Subjects and method:**

262 patients (140, E-HD; 122, C-HD) were subjected for analysis for comprehensive clinical profiles. They were all participating in the above mentioned study, and they had been under the respective HD treatment for 12 consecutive months without hospitalization. Collected data, such as, physical and laboratory examinations, medications, and self-assessment questionnaires on subjective symptoms (i.e., fatigue and pruritus) were compared between the two groups.

**Results:**

In a 12-month period, no clinical relevant differences were found in dialysis-related parameters between the two groups. However, there were differences in the defined daily dose of anti-hypertensive agents, and subjective symptoms, such as severe fatigue, and pruritus, which were all less in the E-HD group. Multivariate analysis revealed E-HD was an independent significant factor for the reduced use of anti-hypertensive agents as well as the absence of severe fatigue and pruritus at 12 months after adjusting for confounding factors.

**Conclusion:**

The data indicates E-HD could have substantial clinical benefits beyond conventional HD therapy, and support the rationale to conduct clinical trials of H_2_ application to HD treatment.

## Introduction

Enhanced oxidative stress and inflammation in patients receiving hemodialysis (HD) treatment play a crucial role in the increased risk of cardiovascular events and mortality [1–3]. Various therapeutic approaches have been investigated to ameliorate this pathology, such as high-dose vitamin E therapy [4], application of endotoxin-free ultra-pure dialysate, and prescription of acetylcysteine [5]. However, clinically available agents to directly suppress inflammation and oxidative stress are still lacking for HD patients [6].

Molecular hydrogen (H_2_) is an inert gas with no known side effects. Recent studies have shown its biological action as an anti-oxidant and anti-inflammatory agent [7]. Administration of H_2_ dissolved in water, by inhalation or intraperitoneally, can suppress oxidative or inflammatory injury resulting from ischemic reperfusion in the brain [8,9], pharmacological intervention [10], and immune rejection post-transplant [11]. H_2_ reacts with hydroxyl radicals [12], and thus could prevent injury caused by radical oxygen species, leading to the suppression of MAPK, MEK-1, NFkB, and caspase-3, 12 [13–16] and subsequent inhibition of apoptosis. Furthermore, we reported on the up-regulation of the anti-oxidative stress regulator Nrf-2 in rats given H_2_ dissolved water [17]. Thus, the application of H_2_ to HD solutions for its anti-oxidative and anti-inflammatory effects represents a unique clinical approach.

Electrolysed water from the cathode provides unique chemical properties, such as alkalinity, low dissolved oxygen, and high dissolved H_2_ [18]. Thus, we developed a novel HD system using water electrolysis to generate water high in dissolved H_2_ [19–21]. Previous pilot studies, including ours, have reported suppressed interleukin-6, hs-CRP, MCP-1, myeloperoxidase (MPO), decreased oxidative injury of red blood cells and lymphocytes, improved redox status of serum albumin, and amelioration of hypertension [19–25]. In reference to these findings, we have started an observational prospective study to compare the outcomes among patients on E-HD and conventional-HD (UMIN000004857).

The present interim analysis aimed to look at whether there are differences between the two groups in respect to comprehensive clinical profiles during the first 12 months observation, in order to explore the possible clinical effect of E-HD.

## Subjects and method

### Interim analysis: Study cohort and patients’ selection

Two hundred sixty-two patients (140 in E-HD; 122 in conventional-HD;C-HD) were subjected for analysis for comprehensive clinical profiles. They were recruited during 2011 to 2012 from patients of 7 dialysis centers in Japan who participated in the “Prospective observational study of the clinical effects of electrolyzed-water hemodialysis (UMIN000004857)”.

Patients were selected according to the following criteria: patients who continued the respective HD treatment (E-HD or C-HD) for 12 consecutive months from the initiation of the study, and were available for full data collection including the self-assessment questionnaire during the 12 month observation. Patients who needed hospitalization, who had developed cancer, those who died during the first 12 months of the study, were excluded from the analysis. As a result, of the initial 308 patients registered, 262 patients were selected for the retrospective analysis (Table 1). All subjects were treated by standard HD schedule (three sessions/week, 4‐5 h/session), using high-performance biocompatible dialysers with fixed QB (200 ml/min) and QD (500 ml/min). Patients who had been treated by vitamin-E coating dialyzer were excluded from this study. At baseline, there were no statistical differences between the groups in BUN reduction rates by HD: 70.8±7.2% in C-HD group, 70.2±6.4% in E-HD group, respectively (p = 0.07).

**Table 1:** Patients demographics.

Characteristic	E-HD	C-HD	*P* Value
N	140	122	
Age (y)	64.8±11.9	67.9±12.0	0.009
Gender, male/female (%)	73/67 (52/48)	72/50 (59/41)	0.319
Dialysis vintage (months)	83 (39, 130)	53 (27, 106)	0.005
Cause of renal failure (DM, (%))	45 (32)	52 (43)	0.096
Comorbidities (CVD, (%))	60 (43)	25 (21)	<0.001
Body weight (post HD, kg)	56.4±10.7	56.4±11.5	0.582
Pre-dialysis SBP (mmHg)	152.8±24.9	154.4±24.7	0.762
Pre-dialysis DBP (mmHg)	79.3±15.2	79.9±13.5	0.834
Post-dialysis SBP (mmHg)	134.6±23.7	143.4±22.2	0.006
Post-dialysis DBP (mmHg)	73.5±13.5	76.4±13.1	0.121
WBC count (/μL)	5852±1765	5493±1712	0.105
Hemoglobin (g/dL)	11.1±1.2	10.7±1.1	0.004
Serum albumin (g/dL)	3.7±0.3	3.7±0.3	0.737
BUN (mg/dL)	69.1±15.7	67.8±15.7	0.248
Creatinine (mg/dL)	10.7±2.9	10.8±2.5	0.680
Beta 2 microglobulin (mg/L)	26.7±6.5	27.9±7.3	0.224
Serum iron (μg/dL)	57.6±21.8	68.0±29.8	0.001
TSAT (%)	22.9±8.3	25.2±10.4	0.050
C reactive protein (mg/dL)	0.10 (0.05, 0.29)	0.10 (0.05, 0.28)	0.702
Fatigue grade (on HD)	2.9±1.1	3.0±1.0	0.561
Pruritus intensity grade	3.3±0.8	3.4±0.82	0.482
Patients on Anti-hypertensive agents (%)	97(69.3%)	91(74.6%)	0.094
Calcium channel blocker	51(36.4%)	70(57.4%)	0.001
Angiotensin II antagonist/ inhibitor	66(47.1%)	63(51.6%)	0.389
DDD of anti-hypertensive agents	1.59±1.21	2.28±1.48	0.001
Patients with ESA (%)	119(85.0%)	100(82.0%)	0.405
Dose of Erythropoetin (IU/week)	3000 (2000, 6000)	2000 (2000, 6000)	0.498
ERI	6.4 (3.8, 9.4)	5.6 (3.5, 12.6)	0.566
Patients with iv iron infusion (%)	33(23.6%)	5(4.1%)	<0.001

Continuous variables are expressed as mean ± SD, or median (interquartile range), as appropriate. E-HD, electrolyzed water HD; C-HD, conventional HD; DM, diabetes mellitus; CVD, cardiovascular disease; SBP, systolic BP; DBP, diastolic BP; WBC, white blood cell; BUN, blood urea nitrogen; TSAT, transferrin saturation; DDD, defined daily doses; ARB, angiotensin II antagonist; ACEi, angiotensin converting enzyme inhibitor; ERI, erythropoietin resistance index; iv, intravenous. Among characteristic, Gender, Cause of renal failure and Comorbidites are compared by chi-squared test, and others are compared by unpaired Student t-test, or Mann-Whitney U test, as appropriate.

In the present study, in order to explore the possible clinical effect of E-HD, patients’ symptoms, such as fatigue, and pruritus, blood pressure, dose of anti-hypertensive agents, anemia, prescription profiles for anemia, were comprehensively compared between the two groups.

#### Frame of original study cohort and design

Briefly, the original study is a prospective observational study, and its objective is to observe the long-term prognosis of patients undergoing treatment with the E-HD system, and to evaluate differences with C-HD system. The study used non-randomized design, and the candidate patients were selected by physician’s decision. In two centers (Kashima Hospital:KH; Iwaki city, Nikko Memorial Hospital:NMH; Muroran city), candidates for E-HD group were selected by chief physicians, and subsequently, matched control patients on C-HD group were selected from the rest of the patients in the respective centers in terms of demographic background, such as, age, and gender. All patients in two centers (Higashi-Muroran Satellite Clinic:HMC; Muroran city, Horai-Higashi Clinic:HHC; Fukushima city) were selected for E-HD group, since the centers were to employ central type E-HD system to replace completely from conventional HD system. While in three centers in which E-HD system was not available (Noboribetsu Memorial Hospital:NH; Noboribetsu city, Tateishi-Jin Clinic:TJC; Tokyo, Gumyoji-Jin Clinic:GJC; Yokohama city), we asked them to register more than one patients as C-HD group, those who were matched for the characteristics of E-HD group patients of above 4 centers in terms of age (±5 years), HD vintage (±3 years), and gender. The primary composite endpoints include all cause of death, and concomitant disease, such as, cardiac disease (heart failure or myocardial infarction requiring hospitalization, coronary artery disease requiring invasive therapy), stroke (symptomatic cerebral hemorrhage or cerebral infarction confirmed by diagnostic imaging), infectious disease (Infection requiring hospitalization), and, obstructive arteriosclerosis requiring leg amputation. Secondary endpoints include number of hospital admissions, patient symptoms (questionnaires: dialysis-related fatigue, pruritus, dialysis-related amyloidosis), and hematology parameters. In this study, those who received on-line hemodiafiltration or combination therapy with peritoneal dialysis, and a potential subject with a serious disease at the time of enrollment, i.e., severe heart failure (New York Heart Association III/IV), severe liver disease, psychological problems, dementia, malignant disease within the previous 3 months, or an evitably poor systemic condition with an evidently poor short-term prognosis, were excluded from this study. The target sample size of the original study (n = 70< each) was based on an estimated event-free rate of 10% differences at 3 years between groups with 1:1 ratio between them, and calculated from the rationale that a statistical power of 90% and the alpha level 0.05, using a two-sided log-rank test.

**Fig 1:**
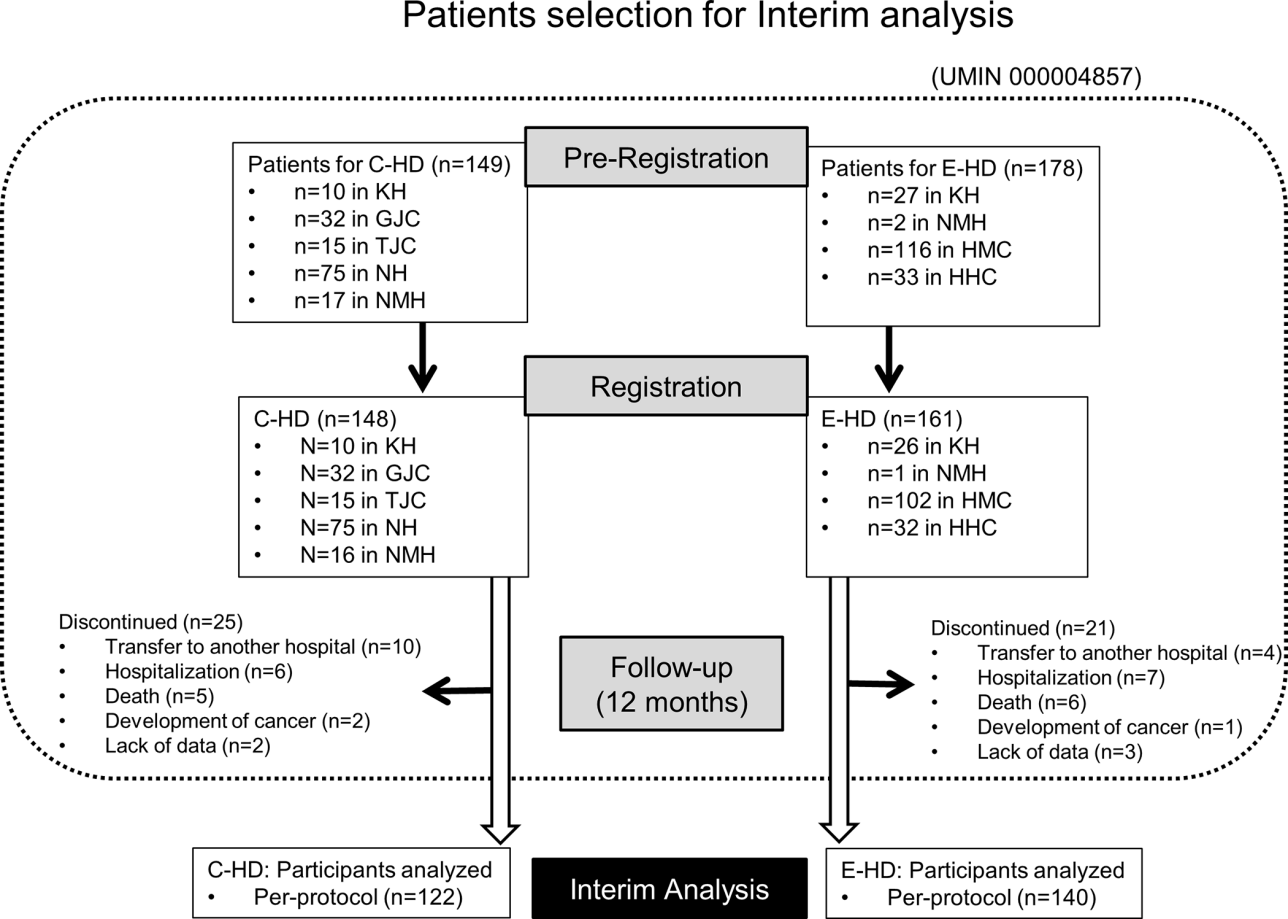
Selection of study participants.

The study was performed according to the open-label design; however, information regarding the potential clinical influences of different dialysis treatments was not provided to the patients. The study was approved by Ethical committee of Fukushima Medical University (No.1155: Supplement 1), and all clinical investigation had been conducted according to the principles expressed in the Declaration of Helsinki. Written informed consents were obtained from all patients registered. As of the end of 2012, 308 patients who gave informed consent to participate in the study, were registered (Fig 1).

### Method

All patients were monitored for subjective symptoms and objective signs during the study period. Blood pressure (BP) was measured using a sphygmomanometer on the upper arm with the patient in a supine position just before starting each HD session, and data were recorded into the clinical record. Administration of iron, erythropoiesis stimulating agents (ESA) to correct anemia, and prescription of agents to control calcium and phosphate, were performed according to the guidelines of the Japanese Society of Dialysis Treatment [26,27]. Prescription of anti-hypertensive agents, and adjustment of body weight after HD (dry weight) were done as needed by the attending physician. Quantities of anti-hypertensive agents were standardized using the defined daily dose (DDD) [28,29].

Regular monitoring of blood was performed at the first HD session of the week (Monday or Tuesday) at least once a month to monitor dialysis status.

Patients were asked to fill out a self-assessment questionnaire every 6 months, which asked about the subjective symptoms of fatigue and pruritus (Table 2).

**Table 2:** Self-assessment questionnaires regarding fatigue and itching.

**I. Fatigue: on dialysis day, off dialysis day**		
**Grade**	**Subjective Level**	**Daily Activities**
Grade 1	Intense fatigue	Disturbed and required rest
Grade 2	Moderate fatigue	Reduced
Grade 3	Mild fatigue	Normal
Grade 4	Tireless	Normal
Grade 5	Inexhaustible	Active
**II. Pruritus**		
**Intensity**		
**Grade**	**Subjective Level**	
Grade 1	Intense	
Grade 2	Moderate	
Grade 3	Mild	
Grade 4	None	
**Frequency**		
**Grade**	**Subjective Level**	
Grade 1	Always	
Grade 2	Sometimes	
Grade 3	Rarely	
Grade 4	None	

All values are expressed as the mean ± standard deviation (SD), or median (interquartile range) as appropriate. For comparisons between two groups, the Student-t test or Mann-Whitney U test was used for continuous variable and chi-square test or Fisher's exact test was used for nominal variable, as appropriate. A within-group comparisons among values at baseline, 6 months and 12 months were analyzed by ANOVA for repeated measures, in the case the distribution of the data was checked for normality prior to applying the repeated measures ANOVA, or Friedman test, as appropriate, with values of P < 0.05 considered statistically significant. Data were statistically analyzed using JMP, version 10.0.2 for Windows (SAS Institute Inc., Cary, NC, USA).

The odds ratio (OR) and 95% confidence interval (CI) for changes in DDD, fatigue, and itching were assessed using multivariate logistic regression analysis with the confounding factors including different patients background between the two groups, such as, age, dialysis vintage, history of CVD, hemoglobin levels.

#### H_2_ deliver HD system

Details of the system have been reported previously [16]. Briefly, test solutions were prepared as follows: pre-filtered water was processed using activated charcoal filtration and water softening to supply the HD-24K water electrolysis system (Nihon Trim, Osaka, Japan), where water was electrolysed by direct current supply to the anode and cathode electrode plates. Water on the anode side was drained, and water from the cathode side (electrolysed water) was collected to supply the reverse osmosis equipment (MH500CX; Japan Water System, Tokyo, Japan) at 500 mL/min. The intensity of electrolysis was adjusted to maintain pH 10.0. The reverse osmosis water made by the water electrolysis system was supplied to make the HD solution. The composition of the inflow H_2_-HD solution was the same as a standard HD solution with the exception of the presence of dissolved H_2_ in the H_2_-HD. The present E-HD system could deliver H_2_ (30 to 80 ppb)-enriched dialysis solution.

## Results

### Changes in the test parameters during the study period Dialysis-related parameters

Between the two groups, statistical differences were found in serum creatinine, and Beta2-microglobulin. However, no clinically relevant significances were found in dialysis parameters, body weigh after HD, serum albumin, and CRP levels (Table 3).

**Table 3:** Changes of clinical and laboratory parameters of the two groups during 12 months.

Variables	Months	E-HD	C-HD	*P* Value
Body weight (kg)	Baseline	56.2±10.5	56.5±11.7	
	6	56.5±10.8	56.5±11.4	
	12	56.6±11.0	56.5±11.9	0.99
Pre-dialysis SBP (mmHg)	Baseline	152.1±24.5	154.4±24.6	
	6	147.1±26.0	147.8±22.7	
	12	152.5±26.0	154.3±20.3	0.96
Pre-dialysis DBP (mmHg)	Baseline	79.1±15.0	80.0±13.6	
	6	76.9±14.7	75.5±14.3	
	12	78.7±14.7	78.2±12.3	0.69
Post-dialysis SBP (mmHg)	Baseline	134.6±23.7	143.4±22.2	
	6	131.2±20.6	140.9±25.7	
	12	134.7±22.0	139.5±21.8	0.19
Post-dialysis DBP (mmHg)	Baseline	73.5±13.5	76.4±13.1	
	6	71.6±12.9	74.2±14.5	
	12	72.9±12.7	73.2±12.7	0.19
WBC counts (/μL)	Baseline	5851.9±1765.2	5492.6±1711.6	
	6	5782.7±1958.4	5437.0±1817.7	
	12	5528.8±1757.8	5400.0±1627.6	0.35
Hemoglobin (g/dL)	Baseline	11.1±1.2	10.7±1.1	
	6	11.0±1.0	10.6±1.2	
	12	10.8±1.2	10.4±1.2	0.82
Serum albumin (g/dL)	Baseline	3.7±0.3	3.7±0.3	
	6	3.6±0.3	3.7±0.3	
	12	3.7±0.3	3.7±0.3	0.11
BUN (mg/dL)	Baseline	69.1±15.7	67.8±15.7	
	6	67.3±16.3	65.0±15.0	
	12	64.9±15.3	66.3±13.7	0.11
Serum creatinine (mg/dL)	Baseline	10.7±3.0	10.8±2.6	
	6	10.6±2.7	11.1±2.5	
	12	10.9±2.8	10.9±2.4	< 0.01
Beta 2 microglobulin (mg/L)	Baseline	26.6±6.3	27.7±7.4	
	6	26.9±5.9	28.2±6.7	
	12	27.6±6.2	27.4±6.5	< 0.01
C reactive protein (mg/dL)	Baseline	0.10 (0.05, 0.29)	0.10 (0.05, 0.28)	0.70
	6	0.11 (0.11, 0.37)	0.09 (0.05, 0.21)	0.08
	12	0.13 (0.13, 0.45)	0.10 (0.05, 0.33)	0.07

Values are expressed as mean ± SD, or median (interquartile range), as appropriate. E-HD, electrolyzed water HD; C-HD, conventional HD; SBP, systolic BP; DBP, diastolic BP; WBC, white blood cell; E, E-HD; C, C-HD.

Changes of C reactive protein are compared in respective months by Mann-Whitney U test, and others are compared between E-HD and C-HD by two-way repeated-measure ANOVA.

#### Anemia and therapy

No clinically significant differences were found in hemoglobin levels (Table 3), iron dose, and ESA dose (data not shown) between the two groups.

#### BP and anti-hypertensive agents

No significant differences were found in SBP/DBP before and after HD between the two groups (Table 3).

Changes of DDD of all patients of respective groups were as follows: 0.64 (0, 1.59) at 0 month, 0.57 (0, 1.18) at 6 months, 0.57 (0, 1.18) at 12 months in E-HD; 1.40 (0, 2.55) at 0 month, 1.33 (0, 2.46) at 6 months, 1.50 (0.03, 2.48) at 12 months in C-HD, respectively. There were significant changes in DDD in E-HD, while, no changes were found in C-HD (p< 0.001, p = 0.093, respectively).

**Fig 2:**
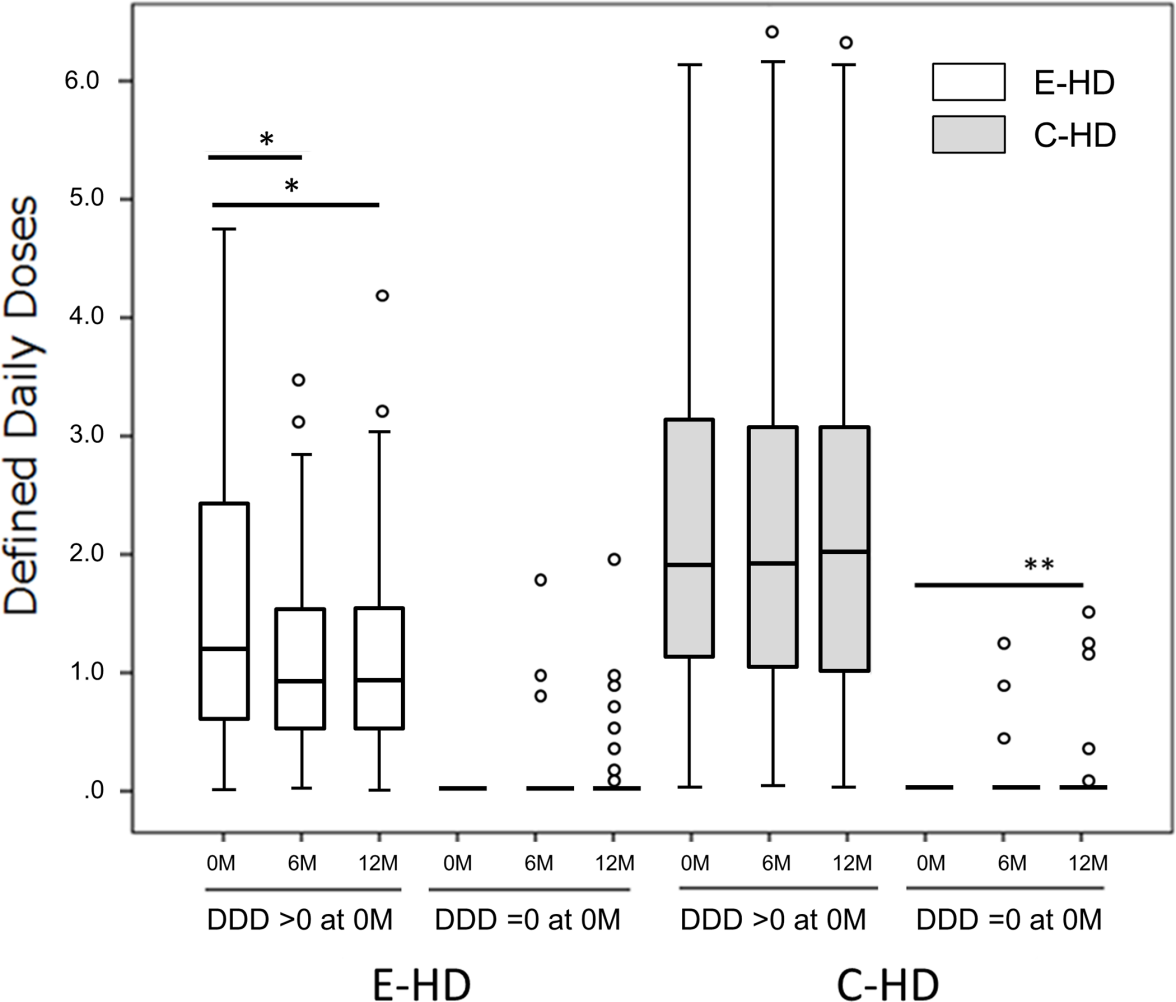
Profiles of the Defined Daily Dose (DDD) of anti-hypertensive agents in the two study groups by function of basal DDD levels. Time course of changes in the Defined Daily Dose of anti-hypertensive agents in the two groups is shown. Each treatment group was further divided into two groups at DDD baseline levels; those of DDD = 0 (43 cases in E-HD, 31in C-HD), and those of DDD >0 (97 cases in E-HD, 91 in C-HD). In cases of DDD = 0, there was a significant change in C-HD, while, no change was found in E-HD, respectively (repeated-measure ANOVA). In cases of DDD >0, there was a significant change in E-HD, while, no change was found in C-HD, respectively (repeated-measure ANOVA). *P<0.001. **P<0.05. E-HD, electrolyzed water HD; C-HD, conventional HD. Circle denotes outliers.

**Fig 3:**
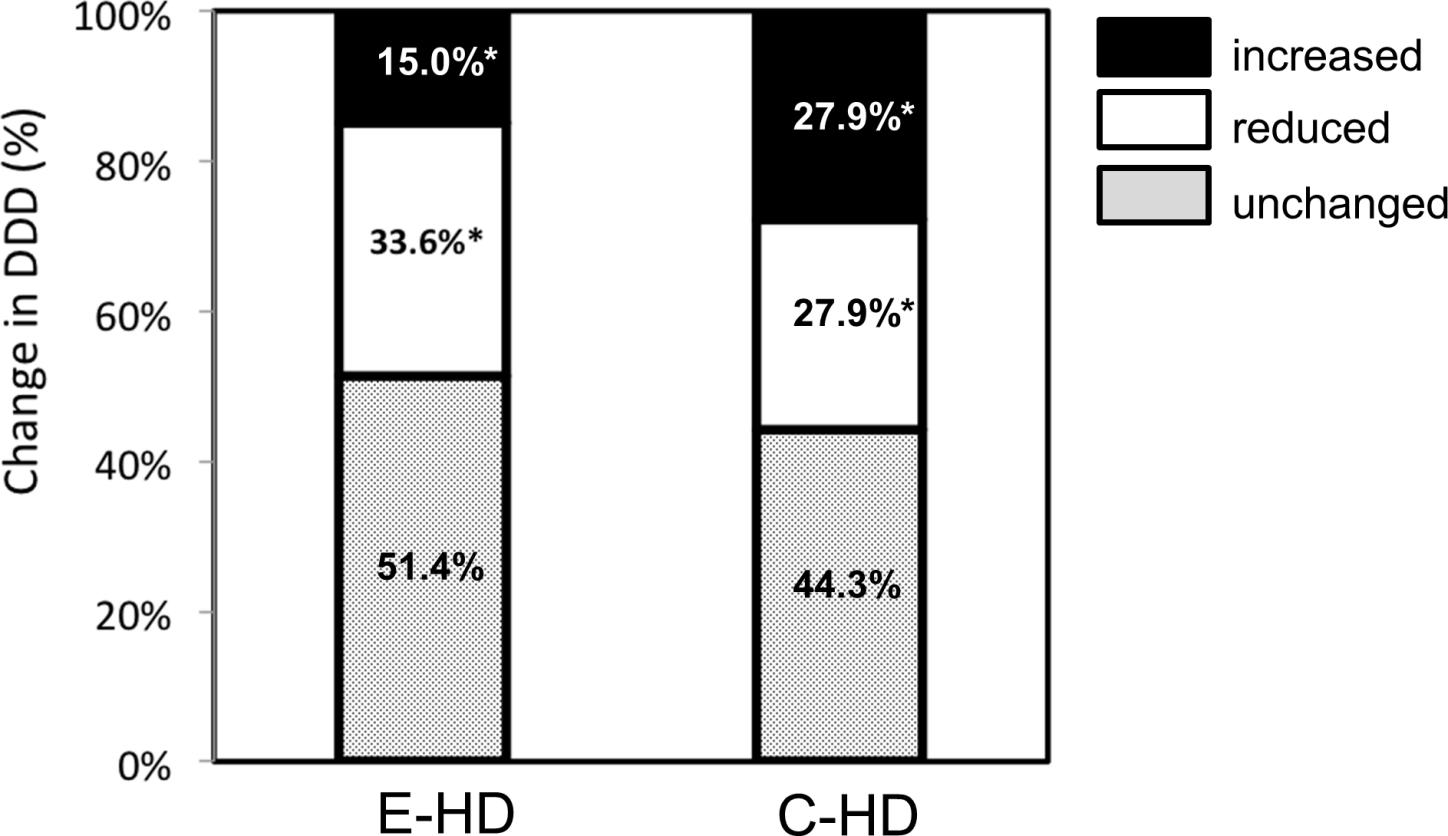
Changes in the defined daily dose of anti-hypertensive agents of the two groups at 12 months. The distribution of changes in the Defined Daily Dose (DDD) of anti-hypertensive agents, categorized as increased, reduced and unchanged, was statistically analyzed using a chi-square test. *P<0.05 E-HD, electrolyzed water HD; C-HD, conventional HD.

DDD analysis is shown according to the prescribed dose (Fig 2); patients who had no medication (DDD = 0 at baseline), and those who had taken medications (DDD >0). In cases of DDD = 0, there were significant changes in C-HD at 12 months as compared to baseline (p<0.05, post hoc test by Tukey method), while, no change were found in E-HD. In cases of DDD >0, there was a significant change in E-HD at 6 and 12 months as compared to baseline (p<0.001, respectively; post hoc test), while, no changes were found in C-HD. At the 12-month point of the study, there were significant changes in the trends of anti-hypertensive agents between the two groups; an increasing number of cases with reduced anti-hypertensive agents was observed (Fig 3).

Multivariate analysis revealed E-HD as a significant factor in reducing the use of anti-hypertensive medications after adjusting for confounding factors (Table 4).

**Table 4:** Influential factors for changes of defined daily dose of anti-hypertensive agents at 12 months.

	Univariate					Multivariate				
Variables	Regression coefficient	Standard error	t value	95%CI	p value	Regression coefficient	Standard error	t value	95%CI	p value
Age	-0.0014	0.0049	-0.29	-0.011 to 0.0083	0.77	-0.0085	0.0056	-1.51	-0.020 to 0.0026	0.13
Male gender	-0.015	0.060	-0.26	-0.13 to 0.10	0.80	0.019	0.058	0.33	-0.095 to 0.13	0.74
Dialysis vintage	0.000071	0.00073	0.10	-0.0014 to 0.0015	0.92	0.000049	0.00075	-0.07	-0.0015 to 0.0014	0.95
DM	0.079	0.061	1.29	-0.042 to 0.20	0.20	0.062	0.061	1.01	-0.058 to 0.18	0.31
Past history of CVD	-0.0046	0.064	-0.07	-0.13 to 0.12	0.94	0.0097	0.060	0.16	-0.11 to 0.13	0.87
Dry weight at baseline	-0.00022	0.0054	-0.04	-0.011 to 0.010	0.97					
Predialysis SBP at baseline	0.0022	0.0024	0.91	-0.0025 to 0.0069	0.37	0.0028	0.0032	0.89	-0.0035 to 0.0092	0.38
Predialysis DBP at baseline	0.0037	0.0041	0.89	-0.0044 to 0.012	0.37	0.0017	0.0057	0.30	-0.0095 to 0.013	0.76
Hemoglobin at baseline	-0.075	0.050	-1.49	-0.17 to 0.024	0.14					
BUN at baseline	-0.0009	0.0039	-0.23	-0.0085 to 0.0067	0.82					
Cr at baseline	0.011	0.022	0.49	-0.032 to 0.053	0.62	-0.016	0.024	-0.67	-0.063 to 0.031	0.51
Serum albumin at baseline	-0.16	0.19	-0.84	-0.55 to 0.22	0.40	-0.11	0.20	-0.53	-0.49 to 0.28	0.59
TC at baseline	-0.00062	0.0017	-0.35	-0.0041 to 0.0028	0.72					
HDL at baseline	-0.0031	0.0027	-1.16	-0.0083 to 0.0022	0.25					
TG at baseline	0.0010	0.00093	1.07	-0.00083 to 0.0028	0.28					
Ferritin at baseline	0.00044	0.00052	0.84	-0.00059 to 0.0015	0.40					
CRP at baseline	-0.036	0.090	-0.40	-0.21 to 0.14	0.69	-0.068	0.081	-0.84	-0.23 to 0.091	0.40
B2M at baseline	0.0045	0.0086	0.52	-0.013 to 0.022	0.60					
DDD at baseline	-0.27	0.037	-7.21	-0.34 to -0.19	<0.01	-0.32	0.038	-8.38	-0.40 to -0.25	<0.01
E-HD	-0.17	0.059	-2.95	-0.29 to -0.057	<0.01	-0.27	0.057	-4.73	-0.38 to -0.16	<0.01

DM, diabetes mellitus; CVD, cardiovascular disease; SBP, systolic blood pressure, DBP, diastolic blood pressure; BUN, blood urea nitrogen; Cr, serum creatinine; TC, total cholesterol; HDL, high density lipoprotein; TG, triglyceride; CRP, C-reactive protein; B2m, B2microglobulin; DDD, defined daily dose of anti-hypertensive agents; E-HD, electrolyzed water hemodialysis.

In multivariate analysis, in addition to factors with p-value less than 0.1 by univariate analysis, clinical-relevant possible influential factors for the prescriptions of anti-hypertensive agents were employed for analysis. Those include dialysis-related factors, such as, age, gender, dialysis vintage, Cr at baseline, cardiovascular and mortality-related factors, such as, presence of DM, history of CVD, nutrition and inflammation (serum albumin and CRP at baseline), and blood pressure-related factors, such as, pre-dialysis SBP and DBP.

### Subjective assessment of fatigue and pruritus

#### Fatigue

**Fig 4:**
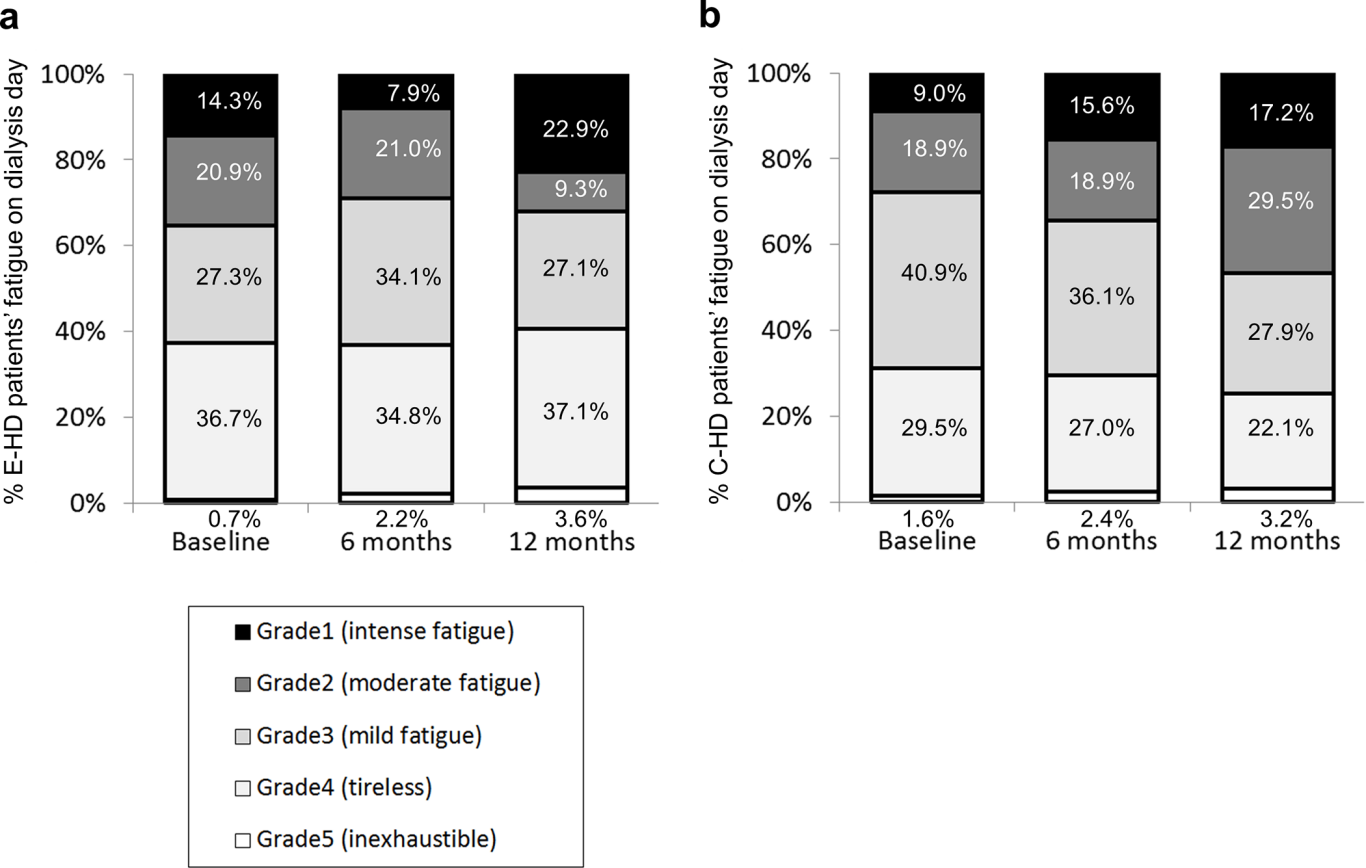
**Profiles of the subjective evaluation of fatigue on dialysis day in E-HD (a) and C-HD (b).** Fatigue symptoms were graded from Grade 1 (most severe) to Grade 5 (no symptoms). No change was found in fatigue profile in E-HD, while, there was a significant change in C-HD (p<0.05) by Friedman test. E-HD, electrolyzed water HD; C-HD, conventional HD.

Fig 4 shows the changes of fatigue grade on dialysis day during 12 months of the two groups. Regarding the changes of grade during the course, no change was found in fatigue profile in E-HD (p = 0.108), while, there was a significant change in C-HD (p = 0.003). In regression analysis, it was revealed that E-HD was a significant negative factor in the presence of severe fatigue (Grades 1+2) at 12 months even after adjusting for confounding factors (Table 5).

**Table 5:** Influencial factors for the presence of severe fatigue (Grade 1+2) at 12 months.

	Univariate			Multivariate		
Variables	OR	95%CI	p value	OR	95%CI	p value
Age	1.029	1.007 to 1.053	<0.01	1.014	0.985 to 1.045	0.35
Male gender	0.91	0.55 to 1.50	0.71	1.14	0.57 to 2.31	0.71
Dialysis vintage	1.001	0.998 to 1.004	0.37	1.005	1.001 to 1.010	0.02
DM	1.53	0.92 to 2.56	0.10	1.61	0.82 to 3.21	0.17
Past history of CVD	1.07	0.63 to 1.81	0.81	0.96	0.46 to 1.99	0.92
Dry weight at baseline	0.99	0.97 to 1.01	0.38			
Predialysis SBP at baseline	0.997	0.987 to 1.007	0.59			
Predialysis DBP at baseline	0.991	0.974 to 1.009	0.33			
Hemoglobin at baseline	0.933	0.755 to 1.151	0.52			
BUN at baseline	0.987	0.970 to 1.003	0.11			
Cr at baseline	0.942	0.860 to 1.031	0.19			
Serum albumin at baseline	0.191	0.076 to 0.455	<0.01	0.566	0.173 to 1.813	0.34
Ferritin at baseline	1.0001	0.9978 to 1.0022	0.94			
CRP at baseline	1.669	1.115 to 2.687	0.01	1.665	1.076 to 2.689	0.02
B2M at baseline	1.043	1.006 to 1.084	0.02	0.995	0.948 to 1.044	0.84
Fatigue day on HD at baseline	8.45	4.74 to 15.53	<0.01	10.03	5.02 to 21.12	<0.01
E-HD	0.54	0.33 to 0.89	0.02	0.30	0.14 to 0.61	<0.01

DM, diabetes mellitus; CVD, cardiovascular disease; SBP, systolic blood pressure, DBP, diastolic blood pressure; BUN, blood urea nitrogen; Cr, serum creatinine; CRP, C-reactive protein; B2m, B2-microglobulin; E-HD, electrolyzed water hemodialysis.

In multivariate analysis, in addition to factors with p-value less than 0.1 by univariate analysis, clinical-relevant possible influential factors for the fatigue were employed for analysis. Those include patients’ basal characteristics, such as, gender, dialysis vintage, and history of CVD.

#### Pruritus

**Fig 5:**
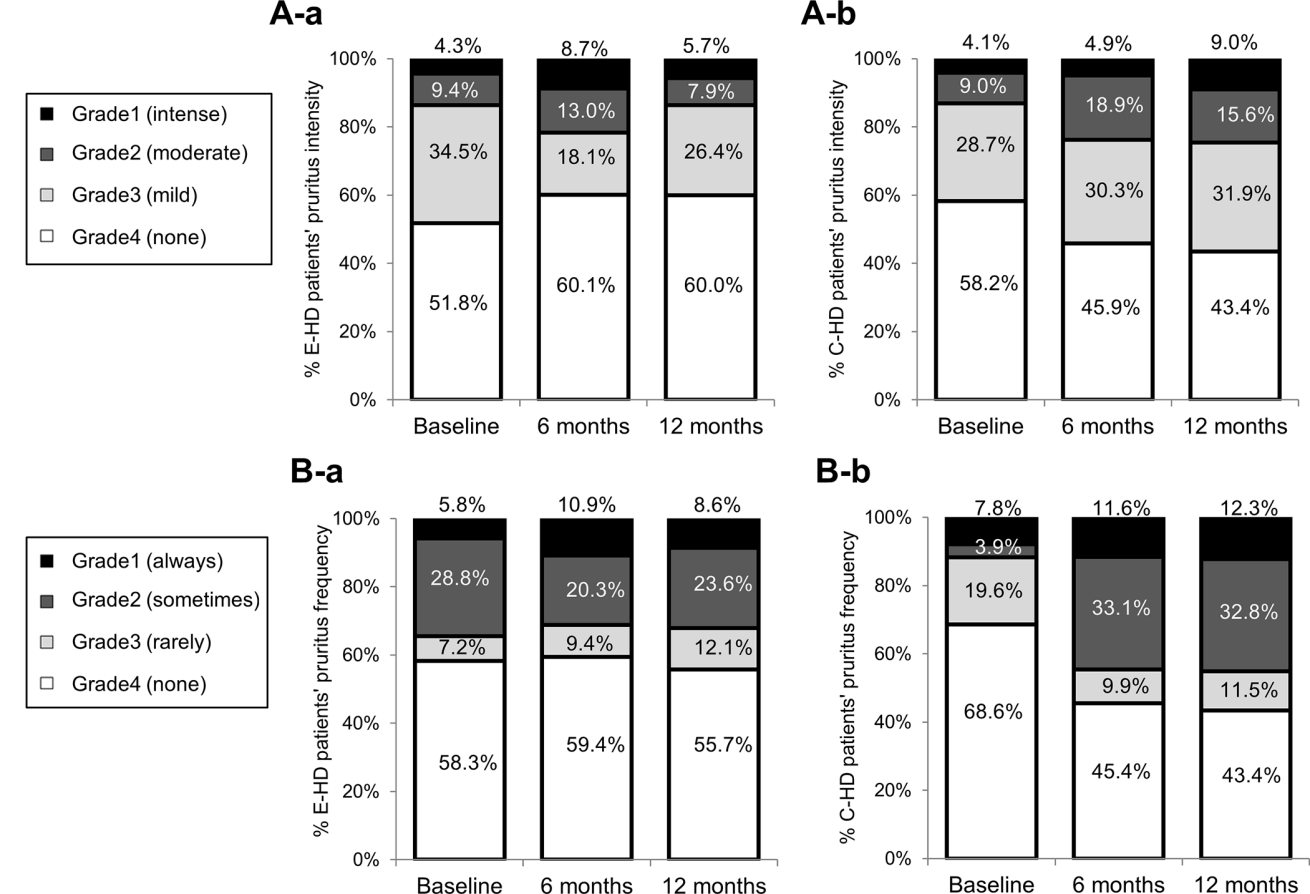
**Profiles of the subjective evaluation of pruritus intensity in E-HD (A-a) and C-HD (A-b), and pruritus frequency in in E-HD (B-a) and C-HD (B-b).** Symptoms were subjectively graded from Grade 1 (most severe) to Grade 4 (no symptoms). No changes were found in pruritus intensity and frequency profile in E-HD, while, there were significant change in C-HD (p<0.05) by Friedman test. E-HD, electrolyzed water HD; C-HD, conventional HD.

Fig 5 shows the changes of pruritus grade of intensity (Fig 5A) and frequency (Fig 5B), respectively, during 12 months of the two groups. Regarding the changes of grade during the course, no changes were found in pruritus intensity and frequency profile in E-HD (p = 0.271, p = 0.609, respectively), while, there were significant change in pruritus intensity and frequency profile in C-HD (p = 0.005, p = 0.002, respectively). In regression analysis, it was revealed that E-HD was a significant negative factor in the presence of pruritus intensity (Grades 1+2), and frequency (Grade 1+2) at 12 months even after adjusting for confounding factors (Table 6).

**Table 6:** Influencial factors for the presence of severe pruritus intensity (Grade 1+2) and severe pruritus frequency (Grade 1+2) at 12 months.

	Univariate			Multivariate		
Variables	OR	95%CI	p value	OR	95%CI	p value
A. Pruritus intensity						
Age	1.013	0.987 to 1.041	0.33	1.008	0.972 to 1.046	0.68
Male gender	0.89	0.48 to 1.67	0.72	0.84	0.39 to 1.83	0.66
Dialysis vintage	1.000	0.996 to 1.004	0.93	1.002	0.997 to 1.008	0.38
DM	1.50	0.79 to 2.81	0.21	1.61	0.74 to 3.56	0.23
Past history of CVD	1.27	0.65 to 2.40	0.48	1.25	0.54 to 2.88	0.59
Dry weight at baseline	1.00	0.97 to 1.03	0.89			
Predialysis SBP at baseline	0.998	0.985 to 1.010	0.72			
Predialysis DBP at baseline	0.983	0.961 to 1.004	0.12			
Hemoglobin at baseline	1.031	0.793 to 1.342	0.82			
BUN at baseline	1.008	0.988 to 1.029	0.44			
Cr at baseline	1.015	0.908 to 1.137	0.79			
Serum albumin at baseline	0.382	0.135 to 1.056	0.06	0.433	0.100 to 1.792	0.25
Ferritin at baseline	1.000	0.997 to 1.003	0.96			
CRP at baseline	0.919	0.499 to 1.440	0.74	0.673	0.269 to 1.328	0.29
Pruritus intensity at baseline	10.61	4.90 to 23.71	<0.01	12.86	5.36 to 32.74	<0.01
E-HD	0.48	0.25 to 0.90	0.02	0.44	0.18 to 0.99	0.047
B. Pruritus frequency						
Age	1.003	0.982 to 1.024	0.79	0.990	0.964 to 1.016	0.44
Male gender	0.92	0.55 to 1.51	0.73	0.85	0.47 to 1.51	0.57
Dialysis vintage	0.998	0.994 to 1.001	0.18	0.998	0.994 to 1.002	0.42
DM	1.62	0.97 to 2.70	0.07	1.72	0.95 to 3.12	0.07
Past history of CVD	1.12	0.66 to 1.90	0.67	1.05	0.56 to 1.95	0.89
Dry weight at baseline	1.00	0.98 to 1.02	0.96			
Predialysis SBP at baseline	0.997	0.987 to 1.007	0.51			
Predialysis DBP at baseline	0.999	0.982 to 1.017	0.94			
Hemoglobin at baseline	1.067	0.864 to 1.320	0.55			
BUN at baseline	1.002	0.986 to 1.019	0.80			
Cr at baseline	1.034	0.945 to 1.133	0.47			
Serum albumin at baseline	0.407	0.173 to 0.928	0.03	0.383	0.129 to 1.096	0.07
Ferritin at baseline	0.998	0.997 to 1.000	0.11			
CRP at baseline	1.035	0.696 to 1.507	0.86	0.946	0.582 to 1.443	0.81
B2M at baseline	1.045	1.007 to 1.086	0.02	1.039	0.995 to 1.087	0.08
Pruritus frequency at baseline	2.80	1.63 to 4.84	<0.01	2.59	1.43 to 4.76	<0.01
E-HD	0.58	0.35 to 0.95	0.03	0.54	0.29 to 0.98	0.041

DM, diabetes mellitus; CVD, cardiovascular disease; SBP, systolic blood pressure, DBP, diastolic blood pressure; BUN, blood urea nitrogen; Cr, serum creatinine; CRP, C-reactive protein; B2m, B2microglobulin; E-HD, electrolyzed water hemodialysis.

In multivariate analysis, in addition to factors with p-value less than 0.1 by univariate analysis, clinical-relevant possible influential factors for the pruritus were employed for analysis. Those include patients’ basal characteristics, such as, age, gender, dialysis vintage, presence of DM, and CRP at baseline (A. Pruritus intensity), and age, gender, dialysis vintage, and CRP at baseline (B. Pruritus frequency).

Regarding dialysis-related amyloidosis, since there were only a few patient in each group, we could not show any substantial data in this interim analysis.

## Discussion

The present study primarily aimed to examine the clinical effects of the addition of H_2_ to HD dialysate. The test dialysate contained an average of 30–80 ppb of H_2_, which was delivered continuously through the dialyzer membrane to the blood during treatment[21]. In the study, all HD systems employed an endotoxin-eliminating filter system. Thus, the different clinical profiles between the two groups, patients on E-HD and those on C-HD, reflects the influence of H_2_ during HD.

During the study, no clinically relevant differences were found in dialysis-related parameters between the two groups. However, there were significant differences between the groups, such as in the required dose of anti-hypertensive agents, in the subjective assessment of severe fatigue and pruritus. Multivariate analysis revealed that E-HD was a significant factor in the reduction of anti-hypertensive agents used, and the absence of fatigue, and the decreased pruritus at 12 months.

### BP and prescribed anti-hypertensives

There were no clinical relevant differences in BP before HD between the groups; however, the required dose of anti-hypertensive agents decreased in E-HD as compared to C-HD, suggesting that E-HD had a mitigating effect on elevated BP. Since there were no significant differences in body weight after HD, the primary mechanism of BP reduction could not be attributed to changes in fluid volume. Rather, it might be related to vasodilation or a reduction in vascular resistance, which needs further study. Ameliorating effects on BP have been observed in previous studies [20,21], and therefore we suppose that H_2_ treatment could potentially benefit patients prescribed multiple medications, by ameliorating BP and reducing the amount or number of required medications.

### Patient-centered outcomes for fatigue and pruritus

**Fatigue**: It has been reported that dialysis-related fatigue is prevalent in 40 to 80% of patients [30–33]. Fatigue reduces activity of daily life (ADL) and quality of life (QOL), and is an independent risk factor for mortality [34]. Multiple factors including psychological factors are involved in dialysis-related fatigue, such as anemia, hemodynamic instability, presence of cardio- and cerebrovascular diseases, nutritional status, and inflammatory conditions accompanying increased levels of hsCRP and IL-6, and TNF-α [35,36]. In the present study, 60% of subjects experienced some level of fatigue on the dialysis day, and 20% on the off dialysis day at baseline. Interestingly, the outcomes of the subjective evaluation of fatigue differed between the two groups. In the multivariate analysis, we employed the clinically relevant confounding factors mentioned above, and E-HD was shown to be a significant factor in the absence of fatigue at 12 months.

**Pruritus**: Pruritus in HD patients reduces ADL and QOL, and is an independent risk factor for mortality [37–39]. In the present study, 40% of the subjects experienced some level of pruritus at baseline. The outcomes of subjective evaluation of pruritus differed between the two groups. In the multivariate analysis, we included possible confounding factors such as calcium, phosphate, dialysis status, and E-HD was revealed to be a significant factor in the absence of pruritus at 12 months.

Taken together, these results lead us to speculate that fatigue and pruritus, at least in part, may be connected to the pathology triggered during HD sessions, and that H_2_ delivery during HD could suppress or ameliorate these symptoms.

The exact mechanisms of the BP reduction and the amelioration of adverse clinical symptoms, such as fatigue and pruritus, by H_2_ delivery, were not revealed in the present study. To date, the exact pathophysiological mechanisms of fatigue and pruritus in dialysis patients have not been clearly defined [36,40]. However, these pathologies reportedly have a connection with enhanced oxidative stress and inflammation in dialysis patients. Specifically, anti-hypertensive agents, such as certain calcium channel antagonist and angiotensin inhibitors, are capable of reducing blood pressure and oxidative stress markers in patients [41]. Furthermore, there are elevated levels of serum IL-6 in patients with fatigue [42], and patients with micro inflammation in uremic pruritus [43,44].

In the present study, there were no differences in % patients on anti-hypertensive agents between the two groups, including angiotensin inhibitors. Therefore, we suppose that H_2_ could play a role in suppressing inflammation, potentially leading to the amelioration of clinical symptoms directly or indirectly related to inflammatory processes. However, there were no significant differences in CRP levels between E-HD and C-HD groups. As for the reasons of lack of association of CRP and clinical presentations, firstly, we speculate that relatively low basal CRP levels of patients in this study may have influenced the result. And secondary, it may due to the possibility that major biological role of H_2_ is to ameliorate the cascade of inflammation, but not the basal inflammatory status of respective patients. This needs to be addressed, along with the exploration of clinically relevant surrogate markers of E-HD, such as, MCP-1, MPO, and albumin redox status, as suggested in the previous studies [21,22].

Although H_2_ appeared to contribute to ameliorating the worsening of patient symptoms, there are several points to be discussed in this study. Firstly, since we aimed to examine the potential clinical effects of E-HD *per se*, we selected subjects from the original observational study cohort (UMIN000004857) to examine only those patients who had undergone the respective HD for 12 months without the episode of hospitalization, or development of *de novo* cancer. As a result, there were several differences in the selected patients’ background of the two groups as shown in Table 1, such as, age, dialysis vintage, history of CVD, and hemoglobin levels. Although E-HD remained significant in the multivariate analysis after adjusting for confounders including those factors, we could not completely exclude the impact of selection bias, and center bias on the result. Secondly, the subjective symptoms were assessed only by self-assessment questioners, and objective data were lacking in the study. Especially, subjective fatigue is closely connected with depression state in some patients. We did not exclude the influence of this point in this study. Thirdly, although the H_2_ levels of dialysates were within 30 to 80 ppb, the levels changed by the season and the facility (data not shown). As to the reason, we suppose the basic water quality and seasonal changes in levels of cationic ions contained in tap water, which could influence intensity of water electrolysis. Thus, the influence of H_2_ levels or its fluctuations in the dialysate on clinical outcomes remains to be elucidated. And lastly, since the present study was an observational one, we could not conclude the clinical effect of E-HD from this study. And randomized clinical study is crucially needed to address this issue.

## Conclusion

The present interim analysis indicate that E-HD has the potential to reduce the required dose of anti-hypertensive agents, and to ameliorate symptoms such as fatigue and pruritus, which all could contribute to the improvement in QOL of chronic dialysis patients. Further study is needed to conclude the clinical impact of the addition of H_2_ to hemodialysis solutions.

## Supporting information

S1 File Study protocol in English. (PDF)

S2 File Study protocol in Japanese. (PDF)

S3 File Trend check list. (PDF)
