# Supplementary material for: Possible clinical effects of molecular hydrogen (H2) delivery during hemodialysis in chronic dialysis patients: Interim analysis in a 12 month observation
Source: PLoS One. 2017 Sep 13;12(9):e0184535. doi: 10.1371/journal.pone.0184535 (PMC5597210; doi:10.1371/journal.pone.0184535)
Supplement: S1 File — (PDF) [file pone.0184535.s001.pdf]

Patient Information Sheet

Request for Participation in a Survey on Electrolyzed-Water Hemodialysis and Standard Hemodialysis

1. Purpose of the survey

   In hemodialysis, blood components come in direct contact with the dialysis solution (dialysate) and dialysis materials, causing chronic stimulation to the patient’s body. This stimulation is believed to cause various complications such as hardening of the arteries (arteriosclerosis). Tohoku University and Nihon Trim Co., Ltd. have jointly developed an electrolyzed-water hemodialysis (EW-HD) system with the aim of reducing this physical stimulation. Previous studies have shown that, like standard hemodialysis, this EW-HD system enables safe dialysis without any side effects. However, we still do not know whether EW-HD has any long-term advantages or effects exceeding those of standard hemodialysis. In this study, we will investigate whether there are any differences in the treatment results of EW-HD and standard hemodialysis

2. Survey details

   The survey will periodically examine the blood test results and symptoms of patients undergoing either EW-HD or standard hemodialysis treatment over the next 5 years. For the blood test, we will perform additional testing on a sample of your blood collected during standard hemodialysis. To investigate your symptoms, we will conduct a short survey about the extent of any fatigue, whole-body itching or pain that you may experience after hemodialysis. We will conduct this survey every 6 months. We will also investigate any episodes of heart disease or stroke and the number of times you are admitted to hospital over the survey period. You will not be required to pay any money for these tests.

3. Risks and disadvantages of this survey

   No major side effects have been confirmed in previous clinical experience with EW-HD. Moreover, the dialysis efficiency of EW-HD is no different to that of standard hemodialysis. The survey data will be handled in a manner that protects your personal information by using a procedure known as “anonymization”. The collected data may also be published at academic conferences or in medical papers in the future. You will not be penalized in any way if you choose not participate in this survey. If you agree to participate, please tell us if you change your mind after the survey has started. You are free to withdraw your consent at any time. We will respect your decision, and your withdrawal will not result in any disadvantage to you in terms of your treatment.

4. Other matters

   Treatment data obtained during the course of this survey will be collected and analyzed by the survey team. This survey teamwill have no involvement in your medical affairs, such as the details of your treatment or your treatment plan, so please talk to your primary physician if you have any questions about your treatment. You will not receive any money for participating in this survey.

Name of medical facility:

Name of principal investigator:

Reference No. 1155
Form 1 (Research purposes)

Application for Approval

Fukushima Medical University
Received on 9 November 2010

Submitted on 9 November 2010

To the Hon.   , Chairman of Fukushima Medical University

I hereby submit this application for approval of the following study protocol with the required forms.

Study title: Prospective observational study of the clinical effects of electrolyzed-water hemodialysis

Study director:

Affiliation: Department of Nephrology, Hypertension, Diabetology, Endocrinology and Metabolism
Name & position:
Tsuyoshi Watanabe, Head of Department
Tel. extension no.: 2320

Department Head Stamp

Attached documents:

1. Study Protocol
2. Request for Study Participation and Study Information Sheet
3. Consent Form
4. Notification of results of COI review stipulated in Article 14-2 of the "Fukushima Medical University Guidelines for Managing Conflicts of Interest" (copy)
5. Other documents (please specify)

* When attaching the "Study Protocol", "Request for Study Participation and Study Information Sheet" and "Consent Form" to this application, use the forms specified by the Ethics Review Board.
* Protection of personal information should be considered before submitting this form and attachments.

Form 2 (Research)

Notification of Application Results

7 January 2011

Department of Nephrology, Hypertension, Diabetology, Endocrinology and Metabolism
To: Professor Tsuyoshi Watanabe

Chairman, Fukushima Medical University

Reference No. 1155

Study title: Prospective observational study of the clinical effects of electrolyzed-water hemodialysis

The results of the review of your application to conduct the above study protocol (changes) are as follows.

Application Review Results

Result	Result of ERB Review	Conditions/details and reasons for required changes
■Permitted<br><br>☐Not permitted<br><br>☐Not applicable	■Approved<br><br>☐Conditional approval<br><br>☐Change(s) recommended<br><br>☐Not approved<br><br>☐Not applicable	

Attached Form 1 (General Ethics Applications and Epidemiology Study)

STUDY PROTOCOL

* Enter the basic information for each item/section of the protocol in concise language and provide any relevant details in numbered references attached to the final page of this application. Where necessary, references should be listed in the protocol body according to their reference number (e.g., Reference 1).

**Study Title**	Prospective observational study of the clinical effects of electrolyzed-water hemodialysis			
**1. Study Personnel (Study director, principal investigator, and investigators)**				
	Name<br>Study personnel taking intramural courses at the university must be indicated by solid boxes (■)		Affiliation	Title
Study director	■	Tsuyoshi Watanabe	Department of Nephrology, Hypertension, Diabetology, Endocrinology and Metabolism	Head of Department
Principal investigator	■	Masaaki Nakayama	Department of Nephrology, Hypertension, Diabetology, Endocrinology and Metabolism	Professor
Investigators (attach as a reference if no. of investigators exceeds 6)	☐	Shigeru Kabayama	Center of Advanced and Integrated Renal Science, Tohoku University	Researcher
	☐	Masato Matsushima	Division of Clinical Epidemiology, Jikei University School of Medicine	Associate Professor
	■	Koichi Asahi	Department of Nephrology, Hypertension, Diabetology, Endocrinology and Metabolism	Lecturer
	■	Yoshimitsu Hayashi	Department of Nephrology, Hypertension, Diabetology, Endocrinology and Metabolism	(Hospital) Lecturer
	☐			
	☐			

2. Type of Study (Check (■) the applicable box)

☐ (1) University research

☐ Collaboration with other facilities (list facilities in section 9. "Study Locations"

☐ No collaboration with other facilities

■ (2) Multicenter study (Attach copies of the ethical review application, protocol, and approval for the relevant clinical protocol of collaborating research organizations)

■ Fukushima Medical University (FMU) is the main study site

☐ FMU is not the main study site

3. **Need for Database Registration** (For registration in a database stipulated in Article 2-2(5) of the "detailed consideration" (limited to those established by the National University Hospital Council of Japan, Japan Pharmaceutical Information Center, and Japan Medical Association), check the relevant box below. If registration is required, indicate the applicable database.)

☐Study for which database registration is mandatory
Database:
■Study for which registration is *not* mandatory
☐Study for which registration is mandatory but prefer not to register → Complete section 4-(4)

4. Desired Review Method (Check the relevant box below)
* The review method listed in (4) below may also be requested only when requesting the method in (1) or (2).

☐ (1) General review

■ (2) Expedited review (Select a reason for the expedited review request by checking the relevant box in a to d below. The description provided in section 10 "Study Methodology" should be worded in a manner that is clearly consistent with the reason selected below.)

☐a. Review of a minor change to a study protocol that has already been approved by FMU's Ethical Review Board (A "minor change" is defined in Article 7-1-1 of the "Ethical Review Board Bylaws of the Fukushima Medical University Ethical Review Board Regulations").

■b. Review of a study protocol not involving more than a minimal risk to subjects (i.e., risks that do not exceed the physical, psychological, or social hazards that could occur in daily life or routine medical tests, and that are of a socially acceptable nature) and satisfying all of criteria i to iv below.

i. A study that involves the collection of data that have already been anonymized in a linkable manner by another organization, a study that involves the conduct an anonymous survey, or a study that does not involve the handling of any personal information.

ii. A study that does not use samples of human origin.

iii.. An observational study that does not involve any physical burden on human subjects.

iv. A survey in which subject responses are voluntary and in which the content of these responses is presumed not to cause any psychological hardship to the subject.

☐c. A study that is limited to the tabulation and simple statistical processing of patient medical records and other medical data held by the medical organization to which the investigators are affiliated.

☐d. A study involving only the commission of data collection or statistical processing based on a contract that includes the following provisions

i. The secure management of data

ii. An obligation of confidentiality

☐ (3) Circulating review (Expedited review may be requested for potentially life-saving studies)

☐ (4) Review requiring Ethical Review Board approval for not registering in a database according to Item (5) of Article 2 "Obligations of the Study Director" in the Ethical Guidelines for Clinical Research.

Reason for application for approval not to register the study in a database:

5. Study Category (Check the relevant box below)

☐(1)Study designated within the scope of the Ethical Guidelines for Clinical Research Check the relevant box below:

☐a.Interventional study relating to the preventive use of drugs or medical devices, or relating to diagnostic or therapeutic techniques (A)

☐b.Interventional study (excluding those that fall under item a above) (B)

■C.Observational study (non-interventional, non-epidemiological study using samples, etc.)

☐Study using samples of human origin:

→☐ Sampling is invasive (C)

→■ Sampling is non-invasive (D)

■Study not using samples of human origin (E)

☐(2)Study falling under the scope of the Ethical Guidelines for Epidemiological Research Check the relevant box below:

☐a.Interventional study

☐Study using samples of human origin:

→☐ Sampling is invasive (F)

→☐ Sampling is non-invasive (G)

☐Study not using samples of human origin:

→☐ Study conducted on individual subjects (H)

→☐ Study conducted on a subject population (I)

☐b.Observational study

☐Study using samples of human origin:

→☐ Sampling is invasive (J)

→☐ Sampling is non-invasive (K)

☐Study not using samples of human origin:

→☐ Study using informational materials not already in existence (L)

→☐ Study limited to the use of existing materials (M)

6. Study Background and Objective (Provide the need, significance of this study, or things to be clarified through this study.)

Background:

The number of end-stage renal failure patients in Japan undergoing chronic dialysis is increasing steadily and has exceeded 280,000 (end of 2008). However, the prognosis of dialysis patients has failed to improve, with an annual mortality rate of 7-8% over the past 10 years. This lack of improvement is linked to the inability to control the leading causes of death in dialysis patients, namely severe cardiovascular and cerebrovascular diseases and infections.

Recent studies have demonstrated that physiological stimuli caused by the dialysis device, dialysis processes, or impurities in the dialysis water (bioincompatibility) increase chronic inflammation and oxidative stress, leading to various complications. The principal investigator (Masaaki Nakayama) has developed a dialysis system using electrolyzed water technology (electrolyzed-water hemodialysis system; EW-HD) and has confirmed that the dialysate produced by this EW-HD system offers good biocompatibility (Industry-academia joint research by Tohoku University School of Medicine and Nihon Trim, Co., Ltd.: Ethical Review Board Approved Study; References 1-3). Based on the results of this research, the research collaborator began marketing a device that produces electrolyzed-water (non-medical device) from July of this year, and use of the device is growing in Japan.

Objective:

To observe the long-term prognosis of patients undergoing treatment with the EW-HD system, and to evaluate differences with conventional hemodialysis systems.

7. Study Population (Provide the rationale and method for selecting study subjects)

Subjects

The study will target patients undergoing chronic hemodialysis treatment with the EW-HD system at medical facilities that have introduced the EW-HD system and patients undergoing chronic hemodialysis treatment with a standard hemodialysis system, and will enroll the patients who provide their informed consent to participate in the study.

EW-HD group: Patients whose primary physician deems them suitable to undergo treatment with the EW-HD system, and patients who wish to undergo treatment with the EW-HD system.

Control group: Patients whose demographic and background factors (sex, age, underlying disease, duration of dialysis) have been matched to those of patients assigned to the EW-HD group.

* Excluded patients:

Patients with a serious disease at the time of enrollment (i.e., patients with an intractable malignant tumor or an evidently poor systemic condition with a very poor short-term prognosis) will be excluded from this study.
Patients under the age of 20 years (minors) and patients with a mental disability or other condition that impairs their decision-making capacity will be excluded from this clinical study.
*Additional matters:
Method of assigning patients to the EW-HD group: To be decided by the study site investigators. FMU will only be responsible for the analysis of anonymized information and will not be involved in the assignment or treatment of patients.
<u>Target sample size:</u>
At least 25 patients in each group
*Rationale for determining patient eligibility:
The target sample size was selected to realize a statistical power of 90% to detect a ±20% difference between groups in the below-mentioned primary composite endpoints.

8. Study Period
Five year-period from December 2010 (scheduled start date) to December 2015

9. Study Locations (Attach references for multiple non-university study sites)
Sites to collect patient data:
Medical institutions (scheduled) to participate in the study
As of 10 November 2010, the following medical institutions have expressed an interest in participating in this study.
Nikko Memorial Hospital (Muroran)
Horai Higashi Clinic (Fukushima)
Yojokai Kashima Hospital (Iwaki)
Mito Chuo Clinic (Mito)

Site to collect anonymized data:
Trim Medical Institute Co., Ltd.
Suite 408, Tohoku University Collaboration Business Incubator (T-Biz) 6-6-40 Aza-Aoba, Aramaki, Aoba-ku, Sendai City, Miyagi 980-8579 Japan
Tel: 022-716-6310 Fax: 022-266-0201

Site to analyze anonymized data:
Medical Office, Department of Nephrology, Hypertension, Diabetology, Endocrinology and Metabolism

10. Study Methodology
Study procedure and organization (Provide a flowchart illustrating the study procedure and organization)
Study group organization, affiliation, and roles:

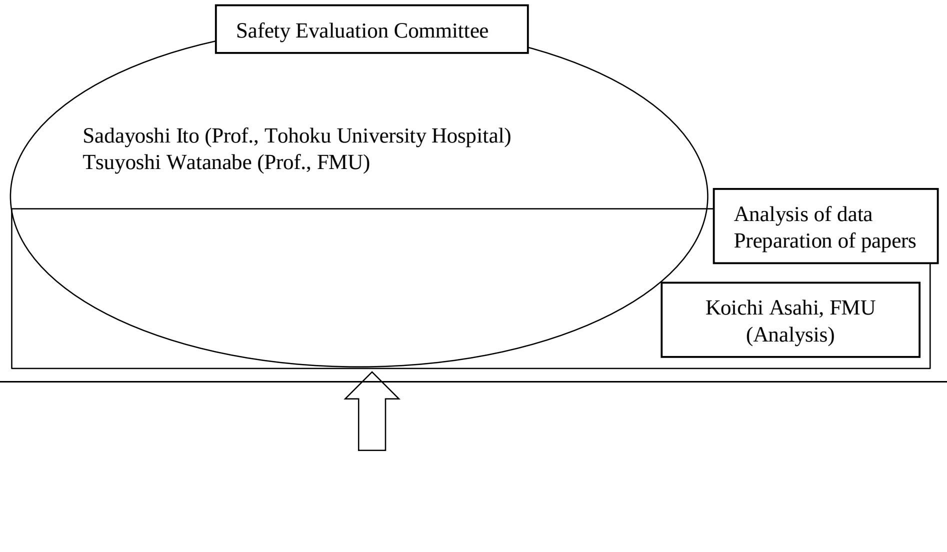

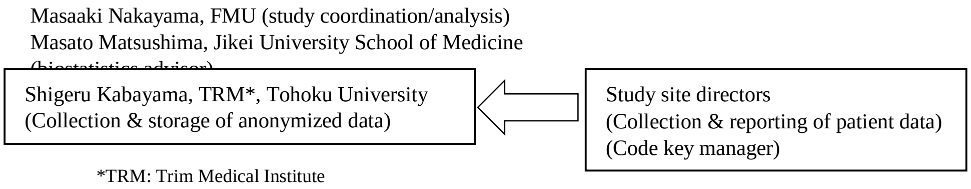

Study procedure:

a. Medical institutions that wish to participate in the study (study sites) will send the signed letter of acceptance to the ERB for approval which will then confirm their eligibility.

b. Study sites will transmit the background and demographic data of enrolled patients (EW-HD and control groups) to the study office in anonymized form. The Study Secretariat will confirm that the patients satisfy the enrollment criteria and will send the study numbers of eligible patients to the study sites.
   - ●After enrollment, the study office will collect medical information based on these study numbers so it will not handle any personal information that could identify the patient.

c. Each study site will enter data on the patients' demographic and baseline characteristics on Survey Forms 1-3 and report the data to the study office every 6 months.
   - ●The study shall continue for the duration of the planned study period.
   - ●The study will be terminated for a patient who dies or is transferred to another facility.
   - ●The study will also be terminated for individual patients who wish to withdraw from the study

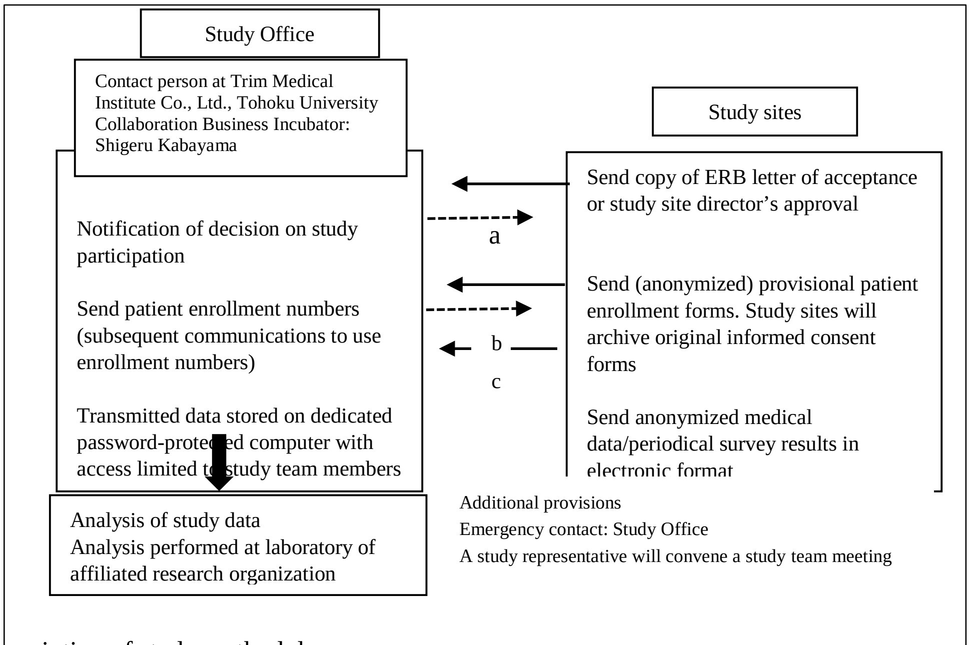

Description of study methodology:

<u>Study period:</u>
Patient enrollment: 2 years from ERB approval
Study period: 5 years from ERB approval

Study sites will be responsible for the above details.

FMU will be responsible for the following study details.
* FMU will not be directly involved in patient recruitment.

FMU will be responsible for monitoring the overall progress of the study, analyzing the anonymized patient data, and preparing papers on the study.
See section “Study procedure” above for details on the handling of anonymized patient data.
The study office (at Trim Medical Institute Co., Ltd., Tohoku University Collaboration Business Incubator) will collect and manage the linkable anonymized data.
The study office will send the data in electronic format to the principal investigator (Nakayama)

- Patient names and numbers and the code key will be managed by the responsible personnel at each study site.
- The study office and researchers in FMU will not handle any information that would enable personal identification.

Study outline:
Observational variables:

- ●Baseline medical information (Survey Form 1)
- ●Primary endpoints:
  1. Total death
  2. Concomitant disease
     a. Cardiac disease: Heart failure or myocardial infarction requiring hospitalization, coronary artery disease requiring invasive therapy.
     b. Stroke: Symptomatic cerebral hemorrhage or cerebral infarction confirmed by diagnostic imaging
     c. Infectious disease: Infection requiring hospitalization
     d. Obstructive arteriosclerosis: Leg amputation
- ●Secondary endpoints:
  1. Number of hospital admissions
  2. Patient symptoms (Survey Form 2)
  3. Hematology parameters (Survey Form 3)

Analysis of the above data variables collected every 6 months after enrollment will be annualized every 12 months.
Between-group differences in major variables will be analyzed for the primary endpoint using Kaplan-Meier analysis (STATA analysis software).

11. Anticipated Study Results and Academic Contribution

It is anticipated that the patients treated with EW-HD will have a better overall prognosis than that of the patients treated with standard hemodialysis.
If the EW-HD system is confirmed to improve prognosis, it will be recognized as a groundbreaking approach to dialysis therapy and will be widely investigated as an original blood purification therapy developed within Japan. It will also enable a broader range of potential medical applications for hydrogen gas, which is one of the features of the EW-HD system, leading to the development of new therapeutics.

12. Storage and Use of Samples (*For studies falling under the "Ethical Guidelines for Epidemiological Research", the terms "samples, etc.", "clinical study" and "subjects" should be replaced by the terms "materials", "epidemiology study" and "study subjects", respectively. However, in item (2), the term "samples, etc." should be read as "samples".).

(1) Method and other details of sample storage (Complete this item where applicable)
Specify the name(s) of sample(s), the storage location, the name of the sample supervisor, the storage method, the storage period, and the timing, method, and anonymization procedure for sample disposal.
Study materials refer to patient medical information collected from medical records.
Storage location: Study Office, Trim Medical Institute Co., Ltd., Tohoku University Collaboration Business Incubator
Storage supervisor: Shigeru Kabayama

Storage method: Electronic media recorded on a stand-alone computer terminal
Storage period: Two years from the end of the study observation period
Method of sample disposal: Destruction of electronic media
Method of anonymization: Study sites will transmit the background and demographic data of enrolled patients (EW-HD and control group patients) to the study office in anonymized form. The study office will confirm that the patients satisfy the enrollment criteria and will send the study numbers of eligible patients to the study sites.
* Handling of samples at FMU: After analyzing the data recorded on the electronic media, FMU will return the data to the study office for destruction.

(2) Use of samples of human origin (Complete this item where applicable)

Researchers/investigators who intend to use samples of human origin collected before the start of the study are generally required to obtain consent to use the samples from the study subjects prior to commencing the study and to create a record of this consent. However, if consent cannot be obtained, the approval of the Ethical Review Board must be obtained when any of the conditions described below in items i. to iii in c. applies. Check the relevant box(es) below.

☐ a. Consent to use samples, etc. has been obtained from subjects, and a record of the consent has been created.

☐ b. Consent to use samples, etc. will be obtained from subjects, and a record of the consent can be created before commencing the study.

☐ c. Although consent to use samples, etc. cannot be obtained from subjects before commencing the study, one of the following criteria applies.

- ☐ i. The relevant samples have been anonymized (here “anonymized” refers to unlinked anonymization or linked anonymization where the researchers/investigators do not possess the code key)
- ☐ ii. Circumstances not covered by i. above, whereby consent that can reasonably be recognized as being substantially related to the objective(s) of the clinical study has been obtained before the start of the study, and information on the study conduct including the intended use of samples, etc. has been made public.
  If this item is applicable, attach reference materials that reasonably demonstrate a substantial relationship between the study objectives and the subject consent and Word file data summarizing the information to be published on the FMU website between the time of study approval and study commencement.
- ☐ iii. Circumstances not covered by i. or ii. above, whereby the criteria listed in 1 to 3 below have been met.
  If this item is applicable, attach reference materials for Word file data summarizing the information to be published on the FMU website between the time of study approval and study commencement (including a description of the purpose of using the samples), and attach reference materials for item 3 below that can be objectively evaluated. In the case of item 2 below, specify this point in the space provided in item (2) “Protection of subject human rights” in section 13 “Ethical Considerations”.
  1. Information on the study conduct including the intended use of samples, etc. has been made public.
  2. Individuals who are scheduled to participate in the study as subjects can refuse to participate.
  3. Where the use of samples, etc. is particularly necessary for improving public health and the consent of subjects is difficult to obtain.

（3）Receipt of samples from other organizations (Complete this item where applicable)

When receiving existing samples, etc. from non-affiliated organization, provide details of the samples, etc. and explain why they are needed.

Patient medical information will be received from an external organization participating in this study.

Details of the samples, etc. to be received: Survey forms 1, 2, and 3 (attached)

（4） Submission of samples, etc. to other organizations (Complete this item where applicable)

As a general rule, when submitting existing samples, etc. to a non-affiliated organization for use in the study, the informed consent of subjects to submit the samples and use them in the study must be obtained, and a record of this consent must be created before the samples can be submitted. However, if the informed consent of subjects cannot be obtained, the samples may be submitted to a non-affiliated organization provided that one of the conditions described below in items i. to iii in c. applies. Check the relevant box(es) below.

☐a. Consent to submit the samples and use them in the study has been obtained from the subjects.

■b. Consent to submit the samples and use them in the study can be obtained from the subjects before they are submitted.

☐C. Consent to submit the samples and use them in the study cannot be obtained from the subjects before they are submitted.

☐ i. The relevant samples have been anonymized (here "anonymized" refers to unlinked anonymization or linked anonymization where the researchers/investigators will not provide the code key)

If some or all of the samples are of human origin, the "Procedure on Submitting Applications, etc. to the Fukushima Medical University Ethical Review Board" (Attachment 2) will be submitted to the head of the affiliated organization before the samples are submitted.

☐ ii. Circumstances not covered by i. above, whereby the criteria listed in 1 and 2 below have been met.

Attach templates of notifications to subjects concerning 1 and 2 below or materials containing any information to be disclosed on the FMU website between the time of study approval and study commencement.

1. The following information on study conduct and the submission of samples has already been communicated or disclosed to subjects.

・That the samples will be used for submission to a non-affiliated organization

・The personal information that will be provided to the non-affiliated organization

・The means and method of submitting the samples to the non-affiliated organization

・That the provision of personal information to the non-affiliated organization that could identify the subject can be terminated at the request of the subject

2. That individuals eligible for enrollment can refuse to participate in the study as subjects.

☐ iii. When submitting information on the health of individuals for use in a socially-significant clinical study, and when alternative suitable measures are to be taken to the extent necessary due to the inability to meet the conditions described in i and ii above due to methodology or content of the clinical study, the content of the health information or some other reason, any information pertaining to items 2-4 in the paragraph marked with an asterisk below must be listed here, or any reference materials pertaining to 2-4 in said paragraph must be attached:

・Reason why the relevant study method will not cause any disadvantage to subjects.

・Reason why the study could not be conducted or the value of the study would be significantly diminished if the relevant study method were not adopted.

・Measures to be adopted in place of informed consent (Check the relevant box below)

☐ The objectives and nature of the sampling and use, including the methods thereof, will be made public to the group to which the subjects belong

☐ A subsequent explanation will be provided to subjects (or to the subject group in the case of an epidemiological study) as soon as possible.

☐ Where samples, etc. are to be collected and used continuously over a prolonged period, an effort will be made to disclose and communicate the situation, including the objectives and methods of sample collection and use, to society.

* Where the submission of samples falls under item iii above, the Ethical Review Board should consider approving the clinical study provided that the study and the submission of samples, etc. satisfies all of the criteria listed in 1 to 5 below.

1. The clinical study does not involve more than a minimal risk to subjects (i.e., risks that do not exceed the physical, psychological, or social hazards that could occur in daily life or routine medical tests, and that are of a socially acceptable nature).
2. The relevant study method will not cause any disadvantage to subjects.
3. The study could not be conducted or the value of the study would be significantly diminished if the relevant study method were not adopted.
4. Either of the following measures can be at taken all times where generally appropriate.
   - The objectives and nature of the sample collection and use, including the methods thereof, will be made public to the group to which the subjects belong.
   - A subsequent explanation will be provided to subjects (or to the subject group in the case of an epidemiological study) as soon as possible.
   - Where samples, etc. are to be collected and used continuously over a prolonged period, an effort will be made to disclose and communicate the situation, including the objectives and methods of sample collection and use, to society.
5. The clinical study has a high level of social significance.

13. Ethical Considerations

(1) Procedure for obtaining informed consent (Check the relevant box below)

■a. The study will be explained using written materials, and informed consent will be obtained in writing.

☐ b. Instead of a written explanation and written consent form, a record of the explanation and the subject’s consent will be created (to be entered and displayed in the subject’s electronic medical record, etc.)

→☐In the case of b. above, state the reason why the relevant method must be used despite the fact that it cannot be used under the ethical guidelines:
Reason:

☐ c. Information on the study conduct will be published because the study does not necessarily require informed consent.

→☐In the case of c. above, state the reason why the relevant method must be used despite the fact that it cannot be used under the respective guidelines:
Reason:

(2) Protection of subject human rights

a. Only individuals who have provided their informed consent will be eligible to participate in the study as subjects.

b. If the eligible individual (subject) or his/her legally-acceptable representative has provided informed consent but later wishes to stop participating in the study once it has started, his/her wishes will be respected.

c. The decision of whether or not to participate in the study is voluntary, and the eligible individual (subject) will not be penalized in any way if he/she decides not to participate or to withdraw from the study.

d. Even after providing informed consent, the eligible individual (subject) or his/her legally-acceptable representative can request an explanation or ask questions about the

study at any time, and will not be penalized in any way for doing so.

e. Patients under the age of 20 years (minors) and patients with a mental disability or other condition that impairs their decision-making capacity will be excluded from this clinical study.

(3) Method for obtaining the informed consent of eligible study participants

Each study site will be responsible for seeking written informed consent after providing a verbal and written explanation of the study. The explanation will cover the information contained in the study protocol submitted to the Ethical Review Board or information based thereupon. Explanatory materials (attached reference pamphlet) will be used to explain the EW-HD system where necessary.

Explanatory reference materials:

Patient Information Sheet

Request for Participation in a Survey on Electrolyzed-Water Hemodialysis and Standard Hemodialysis

5. Purpose of the survey

In hemodialysis, blood components come in direct contact with the dialysis solution (dialysate) and dialysis materials, causing chronic stimulation to the patient's body. This stimulation is believed to cause various complications such as hardening of the arteries (arteriosclerosis). Tohoku University and Nihon Trim Co., Ltd. have jointly developed an electrolyzed-water hemodialysis (EW-HD) system with the aim of reducing this physical stimulation. Previous studies have shown that, like standard hemodialysis, this EW-HD system enables safe dialysis without any side effects. However, we still do not know whether EW-HD has any long-term advantages or effects exceeding those of standard hemodialysis. In this study, we will investigate whether there are any differences in the treatment results of EW-HD and standard hemodialysis

6. Survey details

The survey will periodically examine the blood test results and symptoms of patients undergoing either EW-HD or standard hemodialysis treatment over the next 5 years. For the blood test, we will perform additional testing on a sample of your blood collected during standard hemodialysis. To investigate your symptoms, we will conduct a short survey about the extent of any fatigue, whole-body itching or pain that you may experience after hemodialysis. We will conduct this survey every 6 months. We will also investigate any episodes of heart disease or stroke and the number of times you are admitted to hospital over the survey period. You will not be required to pay any money for these tests.

7. Risks and disadvantages of this survey

No major side effects have been confirmed in previous clinical experience with EW-HD. Moreover, the dialysis efficiency of EW-HD is no different to that of standard hemodialysis. The survey data will be handled in a manner that protects your personal information by using a procedure known as "anonymization". The collected data may also be published at academic conferences or in medical papers in the future. You will not be penalized in any way if you choose not participate in this survey. If you agree to participate, please tell us if you change your mind after the survey has started. You are free to withdraw your consent at any time. We will respect your decision, and your withdrawal will not result in any disadvantage to you in terms of your treatment.

8. Other matters

Treatment data obtained during the course of this survey will be collected and analyzed by the survey team. This survey team will have no involvement in your medical affairs, such as the details of your treatment or your treatment plan, so please talk to your primary physician if you have any questions about your treatment. You will not receive any money for participating in this survey.

Name of medical facility:

Name of principal investigator:

(4) Handling of personal information

Linkable anonymization

The medical information used in the study will include the name of the subject's disease and hematology data. As such, there is a possibility that the data could be entered incorrectly, so linkable anonymization will be performed due to the need for data cleaning during final analysis of suspect data.

Specific method:

The study sites will send the demographic and background data of enrolled subjects in the EW-HD group and control group to the study office in anonymized form. The study office

will then check that the subjects meet the enrollment criteria and will notify the study sites of study numbers of eligible subjects.
The study sites will assign a code or number to each subject's medical information before deleting any personal information and sending the anonymized information as electronic data to the study office.
The study sites will then prepare electronic data on the code key for the assigned symbols or numbers and will securely store the data.

Storage method: The data will be stored in a locked archive under the supervision of the each study site director.

Collection of anonymized data: The study office will manage the anonymized data in electronic format on a stand-alone computer terminal.
Storage managers: Investigator Shigeru Kabayama and Study director Masaaki Nakayama
The collected data will be transferred to principal investigator Massaki Nakayama in electronic format for use in analysis. When using external data storage devices, a non-networked stand-alone computer terminal will be used.
* Handling of data at FMU: After the electronic data have been analyzed, they will be returned to the study office, and any copies will be destroyed.

(5) Potential risks or disadvantages to subjects
Since this study involves the periodic collection and analysis of data every 12 months, the study does not pose any risk to subjects.
All of the patient medical information collected in this study will anonymized, so there is no possibility that personal patient information will be leaked.

(6) Response in the event that the risks described in (5) above actually occur or are expected to occur
If the study results indicate a significant worsening in the prognosis of an EW-HD group patient, the Safety Evaluation Committee will immediately be convened to decide whether to discontinue the study based on a detailed consideration of the results. The committee will promptly notify the study sites of its decision. The Study Group will not be involved in any matters concerning patient compensation.

14. Disclosure of Study Results

(1) Matters in the "Ethical Guidelines for Clinical Research" and "Ethical Guidelines for Epidemiological Research" that must be disclosed to the Ethical Review Board
Check the relevant boxes in a to d below on the matters to be disclosed to the Ethical Review Board, and provide specific details when selecting items ii or iii.

a. Study title
b. Investigators (principal investigator and person providing explanation at ERB meeting)
c. Outline of the meeting stipulated in Article 6-1 of the Fukushima Medical University Ethical Review Board Regulations
d. Results of the study review

■ i .The information specified in a to d above can be disclosed.
☐ ii .Some matters cannot be disclosed under the provisions in Article 2-1-(2)-2 of the Ethical Guidelines for Epidemiological Research.
Matters that cannot be disclosed:
Reason for non-disclosure:
☐ iii .Non-disclosure of part or all of the above matters for reasons other than those covered by ii above
☐ Total non-disclosure:

☐ Partial non-disclosure items:
Reason(s) for partial/total non-disclosure:

(2) Disclosure to media organizations
Check the relevant box below regarding the disclosure of approved study protocol forms and attached documents in response to media inquiries.
■ a. All matters can be disclosed
☐ b. Some matters can be disclosed (Check the items that can be partially disclosed and specify the reason(s) for partial disclosure).
☐ Study title
☐ Investigators
☐ Outline of the meeting stipulated in Article 6-1 of the Fukushima Medical University Ethical Review Board Regulations
☐ Results of the study review
☐ Other matters:
Reason(s) for partial disclosure:
☐ c. Total non-disclosure (Specify reasons).
Reason(s):

(3) Disclosure of information on study subjects (responding to requests from subjects for disclosure of information)
The part of the study being conducted by FMU does not include any personal information that would enable the identification of individual patients, so FMU is unable to provide analytical information on specific patients. Decisions regarding the disclosure of this information will instead be made by the respective study sites.
However, in the event of requests for information on the analytical results of the entire study, the principal investigator Masaaki Nakayama will disclose the annually updated results.

15. Funding

(1) Details of study funding
Funding for the study will be provided as part of the costs of collaborative research by Tohoku University and Nihon Trim Co., Ltd. (Study title: Comprehensive study to inhibit oxidative stress disorders in chronic kidney disease).

(2) Special considerations on conflicts of interest
None.

16. Other Important Matters Related to the Study (Add any relevant information on the study not covered in the above sections)

Sites in Japan that have implemented the EW-HD system as of November 2010:
Nikko Memorial Hospital (Muroran)
Tohoku University Hospital (Sendai)
Katta Hospital (Shiraishi)
Yojokai Kashima Hospital (Iwaki)
Mito Chuo Clinic (Mito)

Survey Form 2-1

Enrollment No.
Hemodialysis: EW-HD / Standard HD

Patient status at time of survey

☐Continuing treatment
☐EW-HD discontinued / interrupted
Reason:
☐Transferred
☐Hospitalized
☐Patient's wishes/circumstances
☐Decision of primary physician
☐Death
Cause of death:

Details of hemodialysis

Duration: hours
Method:
☐HD
☐HDF
☐Online HD
Dialysate:
☐Kindaly
☐Carbostar

Dialysis data (dd/mm/yyyy)

Body weight (kg)<br>Blood pressure (mmHg); Systolic/diastolic<br>Pulse (bpm)<br>CTR (%)	

Prescribed medication

1. Antihypertensive drugs
   ☐No ☐Yes
   Name of prescription drug
2. Vasopressor drugs
   ☐No ☐Yes
3. Antidiabetic drugs (insulin, oral medicine)
   ☐No ☐Yes
4. ESA dosing
   ☐No ☐Yes
   Name of prescription drug
   Dosage × doses/week
5. Administration of iron
   ☐No ☐Yes
6. Antipruritic drugs (oral)
   ☐No ☐Yes
   Name of prescription drug

Concomitant diseases (Start of survey to 6 months)

1. Cardiac disease onset*[1]
   ☐No ☐Yes
   Date of onset (dd/mm/yyyy):
   ☐Congestive heart failure
   ☐Acute myocardial infarction
   ☐Angina pectoris
   ☐Other

2. Stroke onset*[2]
   ☐No ☐Yes
   Date of onset (dd/mm/yyyy):
   ☐Cerebral hemorrhage
   ☐Cerebral infarction
   ☐Other

3. Malignant tumor onset*[3]
   ☐No ☐Yes
   Date of onset (dd/mm/yyyy):
   Disease name:

4. ASO onset (requiring leg amputation)
   ☐No ☐Yes
   Date of onset (dd/mm/yyyy):

5. Infectious disease requiring hospitalization
   ☐No ☐Yes
   Date of onset (dd/mm/yyyy):
   Disease name:

6. Hospitalization for all diseases
   ☐No ☐Yes
   Duration of hospitalization

Definition of onset date:
Date of obvious clinical onset (1 and 2)
Date of test-based diagnosis (1, 2 and 3)
Date of surgery (4) or date of hospitalization for patients in whom surgery is indicated but not feasible (5)

*1: Heart failure or myocardial infarction requiring hospitalization, coronary artery disease requiring invasive therapy.
*2: Symptomatic cerebral hemorrhage or cerebral infarction confirmed by diagnostic imaging
*3: Recurrence of the same type of cancer will be counted as new cancer onset if more than 5 years have elapsed since the end of previous treatment.

Survey Form 2-2

Confirmation of Symptoms

1. Dialysis hypotension
2. Dialysis-related fatigue
3. Whole-body pruritus
4. Dialysis amyloidosis

1. Dialysis hypotension

Definition: Hypotensive patients on hemodialysis who require saline infusion
Method of assessment: No. of days/week on which saline infusion was required: Assess based on previous week
☐1×
☐2×
☐3×

2. Dialysis-related fatigue

Fatigue: on dialysis day, off dialysis day

Grade	Subjective Level	Daily Activities
☐Grade 1	Intense fatigue	Disturbed and required rest
☐Grade 2	Moderate fatigue	Reduced
☐Grade 3	Mild fatigue	Normal
☐Grade 4	Tireless	Normal
☐Grade 5	Inexhaustible	Active

3. Whole-body pruritus

Intensity

Grade	Subjective Level
☐Grade 1	Intense
☐Grade 2	Moderate
☐Grade 3	Mild
☐Grade 4	None

Frequency

Grade	Subjective Level
☐Grade 1	Always
☐Grade 2	Sometimes
☐Grade 3	Rarely
☐Grade 4	None

4. Dialysis amyloidosis

Intensity

Grade	Subjective Level
☐Grade 1	Intense
☐Grade 2	Moderate
☐Grade 3	Mild
☐Grade 4	None

Frequency

Grade	Subjective Level
☐Grade 1	Always
☐Grade 2	Sometimes
☐Grade 3	Rarely
☐Grade 4	None

Survey Form 3

Hematology (dd/mm/yyyy) / /

WBC	g/dl
Hemoglobin	g/dl
Hematocrit	%
BUN	mg/dl
Creatinine	mg/dl
Ca	mg/dl
Phosphorus	mg/dl
Albumin	g/dl
Total cholesterol	mg/dl
HDL cholesterol	mg/dl
Triglycerides	mg/dl
Iron	µg/l
UIBC	µg/l
Ferritin	mg/dl
CRP	mg/dl
ß2 microglobulin	mg/l

Special tests (1)

Methylglyoxal
Lactic acid (pre-/post-dialysis)
hANP
NT-pro BNP
MCP-1
8OHdG
EPC

Special tests (2)

Electrolyzed water/hydrogen concentration
Dialysate/hydrogen concentration
Breath hydrogen concentration before dialysis
Breath hydrogen concentration during dialysis
