# Supplementary material for: Possible clinical effects of molecular hydrogen (H2) delivery during hemodialysis in chronic dialysis patients: Interim analysis in a 12 month observation
Source: PLoS One. 2017 Sep 13;12(9):e0184535. doi: 10.1371/journal.pone.0184535 (PMC5597210; doi:10.1371/journal.pone.0184535)
Supplement: S2 File — (PDF) [file pone.0184535.s002.pdf]

患者説明文

「電解水透析と通常透析の実態調査」　ご協力のお願い

１．調査の目的

血液透析では、血液の成分が透析液や透析機材と直接触れ合うことで、体が慢性的な刺激を受けます。これが動脈硬化など様々な合併症の原因になると言われています。この刺激を軽減する目的で、電解水透析システムが考案されました（東北大学と株式会社日本トリムとの共同開発）。現在までの検討では、このシステムは、通常透析システムと同様に大きな副作用も無く安全に透析が行えることが報告されています。しかし、長期的に、電解水透析システムで通常の透析以上の利点や効能があるかについてはわかっていません。

今回の研究は、電解水透析と通常の透析とで、両者の治療結果に違いがあるかを調べます。

２．具体的な調査内容

電解水透析、通常透析、いずれの治療を受けている患者さんも、今後５年間に亘り、定期的に血液検査と自覚症状を調べさせていただきます。血液検査は、通常の透析時に採血している血液を用いて検査を追加します。自覚症状は、透析後の倦怠感や全身のかゆみ、痛みの程度に関して簡単な質問をいたします。これを、６カ月おきに定期的に行います。また、調査期間中、心臓病や脳卒中の発症の有無や、入院した回数を調査します。これらの検査等で、患者さん御本人に特別の金銭的負担がかかることは一切ございません。

３．調査に伴う危険性や不利益

電解水透析の臨床経験では、特別の副作用は確認されていません。透析自体の効率に関しても、通常の透析と変わりません。調査データは個人名が特定されない状態で扱われます（匿名化）。まとまったデータは、将来的に、学会や医学論文に発表されます。調査に参加することを望まない方に対して、不利益が生じることはございません。また、<u>調査の途中で研究協力を取りやめたい方は申し出てください。いつでもこの同意を取り消すことができます。患者さんの意思が尊重されます。その際、取り消しによる治療上の不利益は一切受けません。</u>

４．その他

本調査は、治療経過データを研究チームが収集し解析を行います。したがって、治療内容や方針等の医学的問題に関しては、調査チームは一切関わりませんので、主治医の先生にご相談ください。調査に参加しても、患者さんに謝金が支払われることはございません。

医療施設名

責任医師名

以上

受付番号 1155

様式第１号(**研究用**)

福島県立医科大学 平成22.11.-9 収受 医大企財第 号

許　可　申　請　書

22年11月　9日提出

福島県立医科大学　学長　様

下記研究計画の実施等に関し、許可を頂きたく必要書類一式を添えて申請します。

研究課題名　電解水透析の臨床効果に関する前向き観察調査

[研究責任者]

所　　属　腎臓高血圧・糖尿病代謝内分泌内科講座

職・氏名　主任教授・渡辺　毅　印

内線電話　２３２０

所属長承認印	（渡辺）

[添付書類]

1　研究計画書

2　研究への参加依頼書並びに研究説明書

3　承諾書

4　「公立大学法人福島県立医科大学利益相反のマネジメントに関する要綱」第１４条第２項に規定する審議結果通知書（写し）

5　その他（具体的に記載してください。）

※添付書類の「研究計画書」、「研究への参加依頼書並びに研究説明書」、「承諾書」については、倫理委員会が定める様式を使用すること。

※各様式及び添付書類を提出する際は、個人情報の保護に留意すること。

様式第２号（**研究用**）

申　請　結　果　通　知　書

平成２３年　１月　７日

腎臓高血圧・糖尿病内分泌代謝内科学講座
教授　渡辺　毅　　　様

福島県立医科大学　学長

受付番号＿＿1155＿＿　課題名　　電解水透析の臨床効果に関する前向き観察調査

上記研究計画（変更）の実施等に関する申請について、下記のとおり結果を通知する。

記

結果	倫理委員会の判定の結果	条件、変更の内容及び理由
許　　可（○）	承　　認（○）	
	条件付承認	
不　許　可	変更の勧告	
	不　承　認	
非　該　当	非　該　当	

別紙様式１（一般倫理申請及び疫学研究申請用）

研 究 計 画 書

※各項目には基本事項を簡潔に記入し、詳細は参考資料として通し番号をつけて、本申請書末尾に添付してください。なお，参考資料は本文中の必要個所に番号で，（参考資料－１）のように示してください。

研究課題名	電解水透析の臨床効果に関する前向き観察調査

1　研究者（研究責任者、主任研究者、分担研究者）

	氏 名（本学の学内講習会の受講者は□を■にしてください）		所 属	職 名
研究責任者	■	渡辺 毅	腎臓高血圧・糖尿病代謝内分泌内科講座	主任教授
主任研究者	■	中山昌明	腎臓高血圧・糖尿病代謝内分泌内科講座	教授
分担研究者（６人以上になる場合は，参考資料として別に添付してください。）	□	樺山繁	東北大学 先進統合腎臓科学コアセンター	研究員
	□	松島雅人	東京慈恵会医科大学 臨床疫学教室	准教授
	■	旭 浩一	腎臓高血圧・糖尿病代謝内分泌内科講座	講師
	■	林 義満	腎臓高血圧・糖尿病代謝内分泌内科講座	（病院）講師
	□			
	□			

2　研究の種類（当該研究について該当するものの□を■にしてください。）

□①　学内研究
　①のうち、□他施設の協力あり（施設名を「９　研究場所」に記載する。）
　　　　　　□他施設の協力なし
■②　多施設共同研究（②の場合、共同する臨床研究機関における当該臨床計画に係る倫理審査の申請書、同審査の計画書及び同審査の承認書の写しを添付すること。）
　②のうち、■主管は本学
　　　　　　□主管は本学以外

3　データベースへの登録の必要性（「臨床研究に関する倫理指針」第２の２(5)の規定されたデータベース（国立大学附属病院長会議、財団法人日本医薬情報センター及び社団法人日本医師会が設置したものに限る。）への登録について、次のうち該当するものの□を■とし、「登録する必要あり」の場合はどのデータベースに登録するか記入してください。）

□登録を義務づけられた研究に該当する
　登録するデータベース名（　　　　　　　　　　　　　　　　　　　）
■登録を義務づけられた研究に該当しない
□登録を義務づけられた研究に該当するが登録しないこととしたい→４(4)を記入

4　希望する審査方法（当該研究について該当するものの□を■にしてください。）

※　(4)は、(1)又は(2)を希望する場合のみ、併せて申請することが出来る項目である。

□(1)　一般審査

■(2)　迅速審査（迅速審査を必要とする理由について、次の①から④のうち該当するものの□を■にしてください。また、「１０　研究方法」欄は、該当する理由と合致することが分かるように記載してください。）

□①　既に本学の倫理委員会の承認を受けた研究計画に関する軽微な変更の審査（軽微な変更とは、「福島県立医科大学倫理委員会規程に基づく倫理委員会内規」第７条第１項第１号に規定するものをいう。）

■②　被験者に対して最小限の危険（日常生活や日常的な医学検査で被る身体的，心理的、社会的危害の可能性の限度を超えない危険であって、社会的に許容される種類のものをいう。）を超える危険を含まない臨床研究計画の審査であって、次のアからエの全ての要件に該当する場合

ア　他の機関において既に連結可能匿名化された情報を収集するもの、無記名調査を行うものその他の個人情報を取り扱わないものであること。

イ　人体から採取された試料等を用いないものであること。

ウ　観察研究であって，人体への負荷を伴わないものであること。

エ　被験者の意思に回答が委ねられている調査であって、その質問内容により被験者の心理的苦痛をもたらすことが想定されないものであること。

□③　研究者等が所属する医療機関内の患者の診療録等の診療情報を用いて、専ら集計、単純な統計処理等を行う研究である場合

□④　次に掲げる事項についての規定を含む契約に基づき、データの集積又は統計処理のみを受託する場合

ア　データの安全管理

イ　守秘義務

□(3)　持ち回り審査（人命に関わる等の理由で審査が急を要する場合）

□(4)　「臨床研究に関する倫理指針」中の第２の「２　研究責任者の責務等」の(5)に規定する、データベースに登録（注）しないことについての倫理委員会の承認を求める審査

【登録しないことに対する承認を申請する理由】

（　　　　　　　　　　　　　　　　　　　　　　　　）

5　研究区分（当該研究について該当となるものの□を■にしてください。）

□(1)　「臨床研究に関する倫理指針」の適用範囲に該当する研究

(1)のうち、該当する研究の□を■にしてください。

□①　介入を伴う研究であって、医薬品又は医療機器を用いた予防、診断又は治療方法に関するもの（A）

□②　介入を伴う研究（①に該当するものを除く。）（B）

■③　観察研究（介入を伴わず、試料等を用いた研究であって、疫学研究を含まないもの）

→□③のうち、人体から採取された試料を用いる場合

→□試料の採取が侵襲性を有する（C）

→□試料の採取が侵襲性を有しない（D）

→■③のうち、人体から採取された試料を用いない場合（E）

□(2)　「疫学研究に関する倫理指針」の適用範囲に該当する研究

(2)のうち、該当する研究の□を■にしてください。
□①介入研究
→□①のうち、人体から採取された試料を用いる場合
→□試料の採取が侵襲性を有する（F）
→□試料の採取が侵襲性を有しない（G）
→□①のうち、人体から採取された試料を用いない場合
→□個人単位で行う研究（H）
→□集団単位で行う研究（I）
□②観察研究
→□②のうち、人体から採取された試料を用いる場合
→□試料の採取が侵襲性を有する（J）
→□試料の採取が侵襲性を有しない（K）
→□②のうち、人体から採取された試料を用いない場合
→□既存資料等以外の情報に係る資料を用いる場合（L）
→□既存資料のみを用いる（M）

6　研究の背景及び目的（研究の必要性、意義、明らかにしたいことを具体的に記してください。）

背景
本邦の末期腎不全による慢性透析患者は増加の一途を辿り、ついに28万人を越えている（2008年末）。一方、透析患者の年間死亡率は過去10年以上に亘り7～8%と予後の改善は認められていない。これには、透析患者の死亡の主因となっている過度な心脳血管疾患や感染症罹患を抑制できないでいる点が関与している。
最近までの検討で、透析機器や治療操作、透析水の不純に伴う生体刺激（生体非適合性）が慢性炎症や酸化ストレス亢進を惹起し、これが種々の合併症罹患の病態に関わっていることが明らかにされている。主任研究者（中山昌明）は、この対策として、水電気分解技術を利用した透析システムを構築し（電解水透析システム）、本システムによって作成される透析液の良好な生体適合性を確認してきた（東北大学大学院医学系研究科と株式会社日本トリムとの産学共同研究：東北大学倫理委員会承認研究：論文資料１－３）。この成果を踏まえて、共同研究先は電解水生成機器（医療機器外）を本年７月から一般販売し、国内で普及しつつある。

目的
電解水透析システムで治療を受ける患者の長期的な予後を観察し、従来のシステムとの違いがあるかを評価する。

7　対象者の選定（選定の理由と方法を記してください。）

対象
電解水透析システムを導入している医療施設において、電解水透析システムで慢性血液透析治療を受ける患者、ならびに、通常システムで慢性血液透析治療を受ける患者を選定し、これらの患者の中で本研究への参加の同意を得た例を研究対象とする。
電解透析群：主治医が電解水透析の適応と考える例、電解透析による治療を患者自らが希望する例。
コントロール群：電解透析割付患者に背景因子（性別、年齢、基礎疾患、透析期間）をマッチングさせた患者。
＊除外例
登録時に重篤な疾患に罹患している例（治療困難な悪性腫瘍罹患例、全身状態が悪く短期的な予後が極めて不良であることが明らかな例は除外する。
２０歳未満の未成年である被験者または精神上の障害などにより、判断能力に支障がある被験者は当該臨床研究から除外される。

＊附則事項
電解水透析システムへの患者割り付け法：本研究へ参加する各医療施設の担当医師が決める。
本学は匿名化情報の解析のみを担当し、患者割付や治療の実施には関わらない。

目標症例数
各群 25 例以上。
＊対象例数の決定理由：
目標患者数は、両群間で後述するプライマリーの複合エンドポイントの違いが 20％前後、パワー90％と想定して設定した。

8　研究期間
２０１０年１２月（予定）～　２０１５　年　１２　月末（５年間）

9　研究場所（学外の場所が多数になる場合は，参考資料として別に添付してください。）
患者データの収集場所：
本研究に参加する医療施設（予定）
2010 年 11 月 10 日時点で研究参加の意思を表明している医療施設は以下のとおり。
日鋼記念病院（室蘭）
蓬莱東クリニック（福島）
養生会かしま病院（いわき）
水戸中央クリニック（水戸）

匿名化データの集積場所：
Trim Medical Institute Co., Ltd. 内
〒980-8579 宮城県仙台市青葉区荒巻字青葉 6－6－40
東北大学連携ビジネスインキュベータ(T-Biz) 408 号
TEL 022-716-6310 / FAX 022-266-0201

匿名化データの解析場所：
本学 腎臓高血圧・糖尿病内分泌代謝内科講座医局内

１０　研究方法

＜手順・相互関係図＞（手順、相互関係が分かるよう流れ図を記載してください。）

研究グループの構成・所属と役割

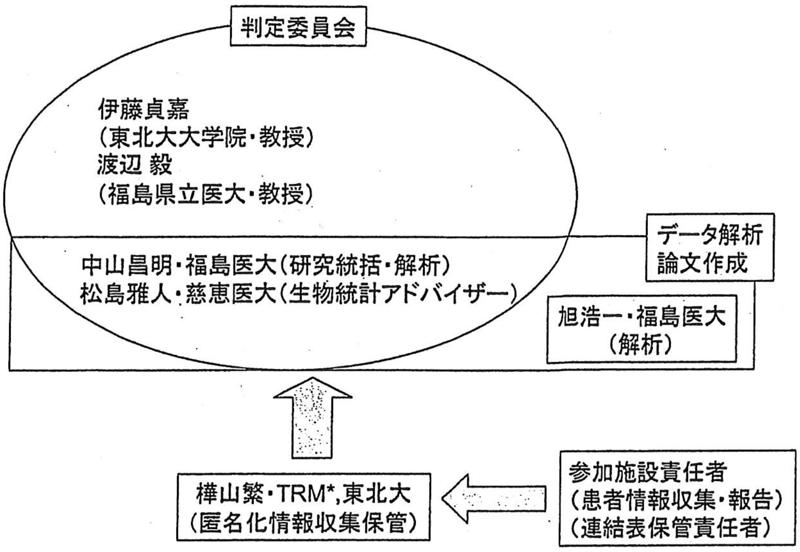

（＊TRM: Trim Medical Institute）

調査の手順

① 研究に参加を希望する施設は、倫理委員会等の承諾書を事務局に送付。事務局から調査受け入れの確認。

② 調査参加施設は、登録患者（電解水透析例とコントロール例）の基礎特性を、匿名化した状態で研究事務局に連絡。事務局は対象例が組み入れ条件に合致しているかを確認のうえ、満たしている場合、調査番号を施設に連絡する。
- 登録後は、事務局では調査番号で医学情報の収集を行う。したがって、調査事務局では、個人が特定できるような個人情報を含むデータは扱わない。

③ 各施設では調査票１，２，３に基礎データを記載し、６ヶ月おきに事務局に報告する。
- この調査を予定された研究期間中、継続する。
- 患者が転院、死亡した場合、調査は打ち切りとする。
- また、患者から研究不参加や中断の申し入れがあった場合も打ち切りとする。

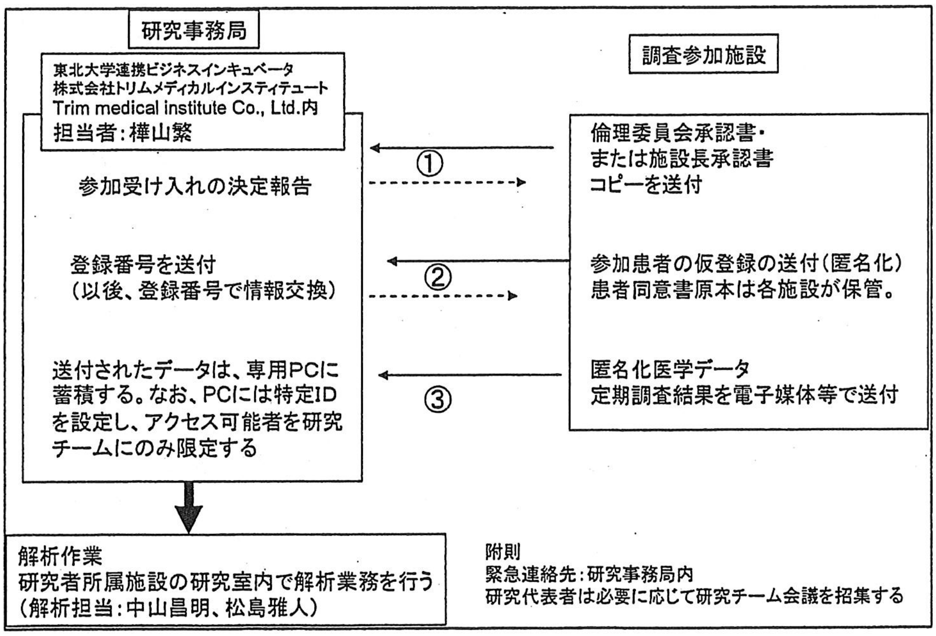

＜研究方法の説明＞

研究期間

患者登録：本学倫理委員会の承認後から２年間
調査期間：本学倫理委員会の承認後から５年間
上記内容に関しては、研究参加施設が担当する。

本学が担当する研究内容

＊本学は患者リクルートには直接関与しない。
本学の担当は、研究全体の進捗状況のモニター、匿名化患者情報の解析と論文化である。
匿名化患者情報の取り扱いは、１０研究方法 調査の手順を参照。
研究事務局（東北大学連携ビジネスインキュベータ内 株式会社トリムメジカルインスティチュートが研究会事務局として匿名化情報（連結可能）を収集・保管する。
これらの情報を電子媒体で主任研究者（中山）に送付する。
＃　患者番号と患者名と連結表は、各医療施設の責任者が管理する。
したがって事務局ならびに本学研究者は個人同定可能な情報は扱わない。

研究概要

観察項目

- 登録時医学情報（調査票１）
- プライマリーエンドポイント：

1. 総死亡
2. 合併症罹患
   ② 心疾患罹患：入院を要する心不全、心筋梗塞、侵襲的治療を要する冠動脈疾患
   ③ 脳卒中罹患：画像上確定した症候性脳出血、脳梗塞
   ④ 感染症：入院を要する感染症
   ⑤ 閉塞性動脈硬化症：下肢切断

- セカンダリーエンドポイント：

1. 入院回数
2. 自他覚症状（調査票２）
3. 各種血液検査（調査票３）

登録後６ヶ月おきに収集するデータを基に、一年おきに上記項目につき解析を行う。
主項目は、カプランマイヤー解析を用いて両群間の差異を統計検討する（解析ソフト：STATA）。

１１　予測される研究結果並びに学術上の貢献

電解透析を受けている患者の全般的な予後が標準透析患者より良好である結果が期待される。
もし、予後改善効果が確認されれば、透析患者にとって、本治療システムは画期的な治療手段として認識され、本邦発のオリジナル浄化療法として広く検討される。さらに、本治療システムの特徴である、水素ガスによる幅広い医療応用への可能性が広がり、新たな治療学を確立することに繋がる。

１２　試料等の保存、利用等について（※この欄について、「疫学研究に関する倫理指針」の適用範囲に該当する研究の場合は、「試料等」を「資料」に、「臨床研究」を「疫学研究」に、「被験者」は「研究対象者」に、それぞれ読み替えてください(ただし、(2)の「試料等」は「試料」と読み替えてください)。）

(1)　試料等を保存する場合の方法等について（該当する場合に記載）

（研究に関する試料等の名称、保管場所、管理責任者、保存方法、保存期間又は廃棄する時期、廃棄する際の方法及び匿名化の方法を記入してください。）

研究資料は、カルテから収集された患者医学情報である。

保管場所：研究事務局（東北大学連携ビジネスインキュベータ内 株式会社トリムメジカルイスティチュート

管理責任者：樺山繁

保存方法：スタンドアローン型PCの電子媒体

保存期間：研究観察期間終了後から２年間

廃棄する際の方法：電子媒体の破壊

匿名化の方法：調査参加施設は、登録患者（電解水透析例とコントロール例）の基礎特性を、匿名化した状態で研究事務局に連絡。事務局は対象例が組み入れ条件に合致しているかを確認のうえ、満たしている場合、調査番号を施設に連絡する。

＊本学での取り扱い：電子媒体でのデータを解析、終了後は研究会事務局に返却・破棄する。

(2)　人体から採取された試料等の利用について（該当する場合に記載）

（研究者等が研究開始前に人体から採取された試料等を利用する場合には、研究開始時までに被験者等から試料等の利用に関する同意を受け、及び当該同意に関する記録を作成することが原則とされていますが、当該同意を受けることができない場合、Ｃの①から③のいずれかに該当することについて倫理委員会の承認を得る必要があります。次のうち該当するものの□を■にしてください。）

□Ａ　試料等の利用に関する被験者等からの同意があり、当該同意に関する記録も作成している。

□Ｂ　研究開始時までに試料等の利用に関する被験者等からの同意を受け、記録も作成できる。

□Ｃ　研究開始時までに、試料等の利用に関する被験者等からの同意を受けられないが、次の①から③のいずれかに該当する。

□①　当該試料等が匿名化（連結不可能匿名化又は連結可能匿名化であって対応表を有していない場合をいう。）されている。

□②　前記①に該当しない場合において、当該臨床研究の目的と相当の関連があると合理的に認められるような同意を研究開始前に受けており、当該臨床研究の実施について試料等の利用目的を含む情報を公開している場合

（なお、これに該当する場合は、許可後から研究開始までに本学ＨＰで情報公開する内容についてまとめたワードのデータ及び被験者等の同意が当該臨床研究の目的と相当の関連があると合理的に認められる参考資料を添付すること。）

□③　前記①及び②に該当しない場合において、次に掲げるアからウの要件を満たしている場合

（なお、これに該当する場合は、許可後から研究開始までに本学ＨＰで情報公開する内容についてまとめたワードのデータ（その中には試料等の利用目的についての記載を含むこと）、及び、ウについて客観的に判断できる参考資料を添付すること。イについては「１３　研究における倫理的配慮について」の「(2)　対象者の人権の擁護」欄にこのことを明記すること。）

ア　当該臨床研究の実施について試料等の利用目的を含む情報を公開していること。

イ　被験者となる者が被験者となることを拒否できるようにすること。

ウ　公衆衛生の向上のために特に必要がある場合であって、被験者の同意を得ることが困難であること。

(3)　**他の機関等から試料等の提供を受ける場合**（該当する場合に記載）
（所属機関以外から既存試料等の提供を受ける場合は、提供を受ける試料等の内容及び必要性を記入してください。）

本研究に参加する外部施設から患者医療情報を受け取る。
収集する内容：調査票１，２，３（添付）

(4)　**他の機関等へ既存試料等を提供する場合**（該当する場合に記載）
（既存試料等の提供を行う者が所属機関外の者に研究に用いるための試料を提供する場合には、試料提供時までに被験者等から試料の提供及び当該研究における利用に係る同意を受け、並びに当該同意に関する記録を作成することが原則とされていますが、当該同意を受けることができない場合には、Cの①から③のいずれかに該当するときに限り、試料を所属機関外の者に提供することができます。次のうち、該当するものの□を■にしてください。）

□A　試料の提供及び当該研究における利用に係る被験者等からの同意がある。
■B　試料提供時までに、試料の提供及び当該研究における利用に係る被験者等からの同意を受けられる。
□C　試料提供時までに、試料の提供及び当該研究における利用に係る被験者等からの同意を受けられない。
□①　当該試料が匿名化（連結不可能匿名化又は連結可能匿名化であって対応表を提供しない場合をいう。）されている場合。
（なお、当該試料の全部又は一部が人体から採取された試料である場合には、試料を提供する前に、所属機関の長に対し「福島県立医科大学倫理委員会への申請等の手順書」別紙２を事前に提出すること。）
□②　当該試料が①に該当しない場合であって、次のア及びイの要件を満たしている場合。
（なお、次のア及びイに関する被験者等への通知のひな形、あるいは、許可後から研究開始までに本学ＨＰで情報公開する内容の資料のいずれかを添付すること。）
ア　当該研究の実施及び試料の提供について以下の情報をあらかじめ被験者等に通知し、又は公開している。
・　所属機関外の者への提供を利用目的とすること
・　所属機関外の者に提供される個人情報の項目
・　所属機関外の者への提供の手段又は方法
・　被験者等の求めに応じて当該被験者が識別される個人情報の研究機関外の者への提供を停止すること
イ　被験者となる者が被験者となることを拒否できるようにしている。
□③　社会的に重要性の高い臨床研究に用いるために人の健康に関わる情報が提供される場合、かつ、当該臨床研究の方法及び内容、当該情報の内容その他の理由により①及び②によることができないため必要な範囲で他の適切な措置を講じる場合に該当する場合。
（なお、※のイ、ウ及びエの内容について次に記載するか、※のイ、ウ及びエの内容について記載された参考資料を添付すること。）
・　当該方法によることが、被験者の不利益とならない理由
（　　　　　　　　　　　　　　　　　　　　　　　　　　　　　　　）
・　当該方法によらなければ、実際上、当該臨床研究を実施できず、又は当該臨床研究の価値を著しく損ねる理由
（　　　　　　　　　　　　　　　　　　　　　　　　　　　　　　　）
・　インフォームド・コンセントに代わる措置の内容（該当するものの□を■にすること。）
□　被験者が含まれる集団に対し、試料等の収集・利用の目的及び内容を、

その方法も含めて広報すること。

□　できるだけ早い時期に、被験者に事後的説明（疫学研究の場合は集団に対するものも可）を与えること。

□　長期間にわたって継続的に試料等が収集又は利用される場合には、社会に、その実情を、試料等の収集又は利用の目的及び方法も含めて広報し、周知される努力を払うこと。

※③に該当する場合、倫理委員会は、当該臨床研究及び試料等の提供が次のアからオの全ての要件を満たしている場合に承認することに留意すること。

ア　当該臨床研究が、被験者に対して最小限の危険（日常生活や日常的な医学検査で被る身体的、心理的、社会的危害の可能性の限度を超えない危険であって、社会的に許容される種類のものをいう。）を超える危険を含まないこと。

イ　当該方法によることが、被験者の不利益とならないこと。

ウ　当該方法によらなければ、実際上、当該臨床研究を実施できず、又は当該臨床研究の価値を著しく損ねること。

エ　一般的に適切な場合には、常に、次のいずれかの措置が講じられること。

・　被験者が含まれる集団に対し、試料等の収集・利用の目的及び内容を、その方法も含めて広報すること。

・　できるだけ早い時期に、被験者に事後的説明（疫学研究の場合は集団に対するものも可）を与えること。

・　長期間にわたって継続的に試料等が収集又は利用される場合には、社会に、その実情を、試料等の収集又は利用の目的及び方法も含めて広報し、周知される努力を払うこと。

オ　当該臨床研究が社会的に重要性が高いと認められるものであること。

１３　研究における倫理的配慮について

(1)　インフォームド・コンセントを受ける手続きについて（該当するものの□を■にしてください。）

■①　文書によって説明し、インフォームド・コンセントは文書で受ける。

□②　文書による説明及び文書による同意に代えて、説明の内容及び被験者から受けた同意に関する記録（電子カルテに入力し表示させる等）を作成する。

→□②のうち、指針上、当該方法によることが出来ないとされている場合であるにも関わらず、当該方法によらざるを得ない場合

その理由（　　　　　　　　　　　　　　　　　　　　　　）

□③　インフォームド・コンセントを必ずしも要しない研究に該当するため、研究の実施についての情報を公開する。

→□③のうち、各種指針上、当該方法によることが出来ないとされている場合であるにも関わらず、当該方法によらざるを得ない場合

その理由（　　　　　　　　　　　　　　　　　　　　　　）

(2)　対象者の人権の擁護

① 本研究は、この研究についての説明を受け、参加することを同意した対象者のみを対象とする。
② 同意を得た対象者（被験者）または代諾者が、本研究の開始後に参加を中止する旨の希望を申し出た場合、その意思を尊重する。
③ 本研究への参加・不参加は、対象者（被験者）の自由意思によるものであり、参加に同意しない場合及び参加を中止した場合でも不利益は受けない。
④ 対象者（被験者）または代諾者は、同意後においても、本研究についての説明を受けまたは質問することができる。またそのことにより、対象者（被験者）は不利益を受けない。
⑤ ２０歳未満の未成年である被験者または精神上の障害などにより、判断能力に支障がある被験者は当該臨床研究から除外される。

(3)　対象者に理解を求めて承諾を得る方法

文書と口頭にて研究内容について説明し、書面による同意書を得る（参加医療施設が独自に行う）。説明する内容は、本倫理委員会提出の研究計画書、あるいはこれに準じた内容を説明する。
電解透析システムについては、資料（参考パンフレット：添付）を必要に応じて用いる。
説明文書の参考文書：

患者説明文

「電解水透析と通常透析の実態調査」　ご協力のお願い

1．調査の目的
血液透析では、血液の成分が透析液や透析機材と直接触れ合うことで、体が慢性的な刺激を受けます。これが動脈硬化など様々な合併症の原因になると言われています。この刺激を軽減する目的で、電解水透析システムが考案されました（東北大学と株式会社日本トリムとの共同開発）。
現在までの検討では、このシステムは、通常透析システムと同様に大きな副作用も無く安全に透析が行えることが報告されています。しかし、長期的に、電解水透析システムで通常の透析以上の利点や効能があるかについてはわかっていません。
今回の研究は、電解水透析と通常の透析とで、両者の治療結果に違いがあるかを調べます。

2．具体的な調査内容
電解水透析、通常透析、いずれの治療を受けている患者さんも、今後５年間に亘り、定期的に血液検査と自覚症状を調べさせていただきます。血液検査は、通常の透析時に採血している血液を用いて検査を追加します。自覚症状は、透析後の倦怠感や全身のかゆみ、痛みの程度に関して簡単な質問をいたします。これを、６カ月おきに定期的に行います。また、調査期間中、心臓病や脳卒中の発症の有無や、入院した回数を調査します。これらの検査等で、患者さん御本人に特別の金銭的負担がかかることは一切ございません。

3．調査に伴う危険性や不利益
電解水透析の臨床経験では、特別の副作用は確認されていません。透析自体の効率に関しても、通常の透析と変わりません。調査データは個人名が特定されない状態で扱われます（匿名化）。
まとまったデータは、将来的に、学会や医学論文に発表されます。調査に参加することを望まない方に対して、不利益が生じることはございません。また、調査の途中で研究協力を取りやめたい方は申し出てください。患者さんの意思が尊重されます。

4．その他
本調査は、治療経過データを研究チームが収集し解析を行います。したがって、治療内容や方針等の医学的問題に関しては、調査チームは一切関わりませんので、主治医の先生にご相談ください。調査に参加しても、患者さんに謝金が支払われることはございません。

医療施設名
責任医師名

以上

(4)　個人情報の取り扱い
う
連結可能匿名化
本研究のために使用する診療情報は、病名や血液検査データを含む。このため、入力や記載データに間違った記入をする可能性がある。これらの疑いデータ値を最終解析の際にデータクリーニングをする必要が生じるため、連結可能匿名化とする。

具体的方法：
具体的には、調査参加施設は、登録患者（電解水透析例とコントロール例）の基礎特性を、匿名化した状態で研究事務局に連絡。事務局は対象例が組み入れ条件に合致しているかを確認のうえ、満たしている場合、調査番号を施設に連絡する。

研究参加施設は、当該診療情報に各個人毎に符号又は番号を振り、そのうえで個人情報を削除して匿名化した電子データを研究会事務局に送付する。

研究参加施設は、符号又は番号の対応表に係る電子データを作成し、それぞれの施設で厳重に保管する。

保管方法：それぞれの施設長の責任の基、研究場所の施錠した保管庫に保管する

匿名化したデータの集積：研究会事務局がスタンドアローン型PC内に電子媒体として保管：
保管責任者：研究分担者（樺山）・研究責任者（中山）

集積されたデータは、解析用資料として電子媒体にて主任研究者の中山に渡される。
なお、当該外部記憶装置を扱う際には、他のコンピュータと切り離されたコンピュータを使用する。
＊本学での取り扱い：電子媒体でのデータを解析、終了後は研究会事務局に返却・破棄する。

(5)　この研究によって生じる可能性のある、対象者にとっての危険性又は不利益事項

本研究は、1 年おきにデータを定期的に収集し、解析を行う。したがって、本研究が患者に危険性を与えることは無い。
また、本研究で収集する患者医学情報はすべて匿名化されたものを扱うため、患者個人情報が漏洩されることは無い。

(6)　上記(5)に記載することが実際に生じた場合、または生じると予知し得た場合の対応

もし、電解水透析患者の予後が有意に不良との結果が確認された場合、ただちに、判定委員会を招集し、結果を詳細に吟味した上で、研究を中止するかを決定する。本結果は直ちに参加施設に伝える。患者補償に関しては、本研究グループは関わらない。

１４　研究内容の公開に関する対応

⑴　「臨床研究に係る倫理指針」及び「疫学研究に関する倫理指針」に規定されている、倫理委員会に対して公表を義務づけられた事項について

（下記の①から④の事項を公表するにあたり、アからウのうち該当するものの□を■とし、イ及びウに該当する場合は具体的な内容を記載願います。）

①　研究課題名

②　研究者氏名（研究責任者及び委員会当日の説明者）

③　福島県立医科大学倫理委員会規程第６条第１項に規定する会議の概要

④　審査結果

■ア　①～④を公表可

□イ　「疫学研究に関する倫理指針」第２の１⑵［２］のただし書き以下に該当するため、一部非公表とする必要がある場合

一部非公表とする事項（　　　　　　　　　　　　　　　　）

非公表とする理由（　　　　　　　　　　　　　　　　）

□ウ　上記イに該当する場合以外で、非公表とする場合又は一部非公表とする場合

□全て非公表の場合

□一部非公表の場合、その事項（　　　　　　　　　　　　　　　　）

（一部）非公表とする理由（　　　　　　　　　　　　　　　　）

⑵　報道機関等に対する公開について（照会があった場合に、承認された研究計画書等各様式及び添付書類の公開について、該当する□を■にしてください。）

■①　全面的に公開して良い。

□②　部分的に公開して良い。（公開してよいものの□を■とし、部分的な公開とする理由を具体的に記載してください。）

□課題名

□研究者氏名

□会議福島県立医科大学倫理委員会規程第６条第１項に規定する会議の概要

□審査結果

□その他（　　　　　　　　　　　　　　　　）

（理由：　　　　　　　　　　　　　　　　）

□③　一切公開しない。（理由を記してください。）

（理由：　　　　　　　　　　　　　　　　）

⑶　対象者に関する情報開示について（御本人から情報開示の請求希望があった場合の対応）

福島県立医大が担当する研究内容には、患者の同定が可能な個人情報は含んでいないため、県立医大から特定の患者の解析情報を提示することは不可能である。本件は、研究に参加している各医療施設の判断において処理される事項となる。

ただし、研究全体の解析結果については、情報開示の希望があった場合、年次ごとの最新結果を主任研究者の中山が開示する。

１５　研究資金の調達方法

(1)　資金についての詳細事項

東北大学と日本トリム株式会社との共同研究に関わる研究費（研究課題名：慢性腎臓病の酸化ストレス障害を抑制するための包括的研究）から拠出する。

(2)　利益相反についての特記事項

特に無し。

１６　その他本研究に関する特記事項（研究の内容上，上記以外で必要な事項があればそれらを追加してください。）

2010 年 11 月時点で、本システムを導入している国内施設：
日鋼記念病院（室蘭）
東北大学病院（仙台）
公立刈田病院（白石）
養生会かしま病院（いわき）
水戸中央クリニック（水戸）

調査1

患者基礎特性
施設

氏名　　　　○○(匿名)
生年月日
性別
体重(基礎体重)
身長
腎臓基礎疾患(原発性腎疾患, 腎硬化症、糖尿病性腎症、その他)
透析開始年度
透析スタイル(HD, HDF, オンラインHD)
透析液(キンダリー、カーボスター)

糖尿病治療の有無(インスリン、内服薬)
血圧治療の有無(降圧薬、昇圧薬)
合併症
心疾患　　心筋梗塞、狭心症、弁膜症、心筋症、その他
脳卒中　　脳出血、脳梗塞、その他
悪性腫瘍　治療中、治療終了
ASO　　　下肢切断の有無
透析合併症(透析アミロイドーシス、透析低血圧、掻痒症、透析後倦怠感)

血液検査(直近データ)
ヘモグロビン
BUN
クレアチニン

調査票2

登録番号
透析:電解水透析 ・ 通常透析

調査時の状況

□ 継続治療中
□ 電解水透析の中止・中断<br>理由:□転院 □入院 □患者希望・都合 □主治医の判断
□ 死亡<br>死因:

透析内容

透析時間:		hr
透析法:	□ HD □HDF □オンラインHD	
透析液:	□キンダリー □カーボスター	

透析データ(年 月 日)

	HD前	HD後
体重(kg)		
血圧(mmHg) ・収縮/拡張	/	/
脈拍(回/分)		
CTR(%)		

処方

1. 降圧薬	□無 □有<br>処方薬名
2. 昇圧薬	□無 □有
3. 糖尿病治療薬(インスリン、内服薬)	□無 □有
4. ESA投与	□無 □有<br>処方薬名:<br>投与量: x 回/週
5. 鉄剤投与	□無 □有
6. 掻痒症治療薬(内服)	□無 □有<br>処方薬名:

合併症の発症 （開始時から6ヶ月目までの期間）

1. 心疾患の発生（注1）	□無 □有	発生日 年 月 日<br>□ うっ血性心不全<br>□ 急性心筋梗塞<br>□ 狭心症<br>□ その他 （ ）	
2. 脳卒中の発生（注2）	□無 □有	発生日 年 月 日<br>□ 脳出血<br>□ 脳梗塞<br>□ その他 （ ）	
3. 悪性腫瘍の発生（注3）	□無 □有	発生日 年 月 日<br>病名 （ ）	
4. ASO（下肢切断対象例）の発生	□無 □有	発生日 年 月 日	
5. 入院を要する感染症の発生	□無 □有	発生日 年 月 日<br>病名 （ ）	
6. すべての入院の有無	□無 □有<br>入院期間		

発生日の定義
臨床的に明らかに発症した日（1，2の場合）
検査で明確に診断された日（1，2，3の場合）
手術を実施した日（4の場合）、あるいは手術適応であっても手術が困難な例では入院日を発生日とする。
入院した日（5の場合）

注1： 入院を要する心不全、心筋梗塞、侵襲的治療を要する冠動脈疾患
注2： 画像上確定した症候性脳出血、脳梗塞
注3： 同一癌の既往歴がある場合、治療終了後5年以降の発生をカウントする。

自他覚症状の確認

1. 透析低血圧
2. 透析関連倦怠感
3. 全身の掻痒感
4. 透析アミロイドーシス

1. 透析低血圧
定義：生理食塩水の投与を要する透析中の血圧低下例
評価法：生理食塩水の投与を要した日数/週 ：直近の前週で評価する
□ 1回
□ 2回
□ 3回

2. 透析関連倦怠感

評価法：患者アンケート
透析後・透析日翌日の自覚症状について
- □ Grade 1：疲れがひどくてしばしば横になる。
- □ Grade 2：少し活動したり動いたりしただけで疲れを感じる。
- □ Grade 3：普通に活動するが体全体の疲れを感じる。
- □ Grade 5：調子は良く活動的である。

3. 全身掻痒感

評価法：患者アンケート
強度の自覚症状について自覚症状について
- □ Grade 1：強度。
- □ Grade 1：強度。
- □ Grade 3：軽度。
- □ Grade 4：なし

頻度の自覚症状について
- □ Grade 1：しょっちゅう。
- □ Grade 2：時々。
- □ Grade 3：たまに。
- □ Grade 4：なし。

4. 透析アミロイドーシス

評価法：患者アンケート
痛みの自覚症状について
- □ Grade 1：強度。
- □ Grade 1：強度。
- □ Grade 3：軽度。
- □ Grade 4：なし

頻度の自覚症状について
- □ Grade 1：しょっちゅう。
- □ Grade 2：時々。
- □ Grade 3：たまに。
- □ Grade 4：なし。

調査表3

血液検査（　年　月　日）

WBC	
ヘモグロビン	g/dl
HT	%
BUN	mg/dl
ceatinine	mg/dl
Ca	mg/dl
リン	mg/dl
アルブミン	g/dl
総コレステロール	mg/dl
HDLコレステロール	mg/dl
中性脂肪	mg/dl
鉄	μg/l
UIBC	μg/l
フェリチン	mg/dl
CRP	mg/dl
β2ミクログロブリン	mg/l

特殊検査①

メチルグリオキサール	
乳酸（透析前・後）	
hANP	□
NT-pro BNP	□
MCP-1	□
8OHdG	□
EPC	□

特殊検査②

電解水・水素濃度	□
透析液・水素濃度	□
透析前・呼気水素濃度	□
透析中・呼気水素濃度	□
